# Virus-mediated suppression of host non-self recognition facilitates horizontal transmission of heterologous viruses

**DOI:** 10.1371/journal.ppat.1006234

**Published:** 2017-03-23

**Authors:** Songsong Wu, Jiasen Cheng, Yanping Fu, Tao Chen, Daohong Jiang, Said A. Ghabrial, Jiatao Xie

**Affiliations:** 1 State Key Laboratory of Agricultural Microbiology, The Provincial Key Lab of Plant Pathology of Hubei Province, College of Plant Science and Technology, Huazhong Agricultural University, Wuhan, China; 2 Department of Plant Pathology, University of Kentucky, Lexington, Kentucky, United States of America; University of California, UNITED STATES

## Abstract

Non-self recognition is a common phenomenon among organisms; it often leads to innate immunity to prevent the invasion of parasites and maintain the genetic polymorphism of organisms. Fungal vegetative incompatibility is a type of non-self recognition which often induces programmed cell death (PCD) and restricts the spread of molecular parasites. It is not clearly known whether virus infection could attenuate non-self recognition among host individuals to facilitate its spread. Here, we report that a hypovirulence-associated mycoreovirus, named Sclerotinia sclerotiorum mycoreovirus 4 (SsMYRV4), could suppress host non-self recognition and facilitate horizontal transmission of heterologous viruses. We found that cell death in intermingled colony regions between SsMYRV4-infected *Sclerotinia sclerotiorum* strain and other tested vegetatively incompatible strains was markedly reduced and inhibition barrage lines were not clearly observed. Vegetative incompatibility, which involves Heterotrimeric guanine nucleotide-binding proteins (G proteins) signaling pathway, is controlled by specific loci termed *het* (heterokaryon incompatibility) loci. Reactive oxygen species (ROS) plays a key role in vegetative incompatibility-mediated PCD. The expression of G protein subunit genes, *het* genes, and ROS-related genes were significantly down-regulated, and cellular production of ROS was suppressed in the presence of SsMYRV4. Furthermore, SsMYRV4-infected strain could easily accept other viruses through hyphal contact and these viruses could be efficiently transmitted from SsMYRV4-infected strain to other vegetatively incompatible individuals. Thus, we concluded that SsMYRV4 is capable of suppressing host non-self recognition and facilitating heterologous viruses transmission among host individuals. These findings may enhance our understanding of virus ecology, and provide a potential strategy to utilize hypovirulence-associated mycoviruses to control fungal diseases.

## Author summary

Self/non-self recognition, as the most fundamental immune system, is an essential part in response to nonself host defense in fungi, plants and animals against pathogens or molecular parasites. Vegetative incompatibility as a conspecific nonself system in filamentous fungi results in PCD when cells of incompatible strains contact each other, which also restricts the spread of molecular parasites. The hypovirulence-associated Sclerotinia sclerotiorum mycoreovirus 4 (SsMYRV4), was found to function as a potent inhibitor of G protein signaling pathway, ROS production and vegetative incompatibility-mediated PCD. This SsMYRV4-mediated unique feature enables horizontal transmission of heterologous mycoviruses among vegetatively incompatible *S*. *sclerotiorum* individuals in an interaction system (SsMYRV4/*S*. *sclerotiorum*) to create a bridge donor strain for mycovirus spread under natural conditions. Furthermore, this virus-mediated suppression of host non-self recognition provides a potential strategy for virus spread that can enhance our understanding of virus ecology.

## Introduction

Non-self recognition is a universal phenomenon among all organisms [1–4]. Organisms may recognize non-self RNA, DNA or proteins of pathogens and parasites. Non-self recognition often occurs at an early stage of invasion by parasites and may initiate strong counteraction of the host against the infection by parasites. The counteraction may include, but is not limited to, innate immunity, hypersensitive reaction and RNA interference [4–8]. Non-self recognition as a fundamental attribute of cell-cell interactions also occurs widely among organisms. Bacteria have a contact-dependent growth inhibition system to inhibit the growth of the same species [9]; flowering plants discriminate between self and non-self pollen to prevent inbreeding [10]. Non-self recognition is often a major limiting factor for successful organ transplantation [11], and spread of viruses among individuals [12].

Generally, non-self-recognition, which is vegetative incompatibility or heterokaryon incompatibility in fungi, is controlled by several heterokaryon/vegetative incompatibility (*het*/*vic*) genes [13]. There are nine, eleven, and six *het* loci, in *Podospora anserina* [14], *Neurospora crassa* [15] and *Cryphonectria parasitica* [16], respectively. Heterotrimeric guanine nucleotide-binding proteins (G proteins) as universal signaling proteins in eukaryotes are involved in nutrient-sensing, pheromone response/mating, pathogenesis and non-self-recognition pathways in filamentous fungi [17]. The α subunit of G proteins from *P*. *anserina*, has been confirmed to be involved in both regulation of development and vegetative incompatibility [18]. With a few exceptions [19], non-self recognition of fungi often leads to strong reactions of contacting cells, such as programmed cell death (PCD) [20]. Thus non-self recognition was considered as an important barrier to protect the spread of molecular parasites including mycoviruses [12, 13], and it was considered to be evolutionarily created due to pathogen defense [21]. Non-self recognition of fungal individuals is a limiting factor for the spread of parasites whose horizontal transmission is dependent on somatic fusion [13, 22–26]. However, whether viruses or other parasites could suppress non-self recognition among fungal individuals has not been investigated.

Mycoviruses, also called fungal viruses, are widespread in all major groups of fungi [27]. The diversity of mycoviruses is markedly high; mycoviruses are grouped into at least twelve families with many mycoviruses remaining unclassified [27]. Aspergillus fumigatus tetramycovirus-1 appears to be an intermediate between double-stranded (ds) RNA and positive-stranded (+ss) RNA viruses, as well as between encapsidated and capsidless RNA viruses [28]. Furthermore, many novel mycoviruses were discovered frequently by deep sequencing analysis of fungal RNA samples [29]. To date, mycovirus infections have been reported to reveal various types of interaction relationships between heterologous viruses and host fungi [8, 30]. Thus discovery of new mycoviruses may provide clues for studies on ecology and evolution of viruses [31–33]. Furthermore, hypovirulence-associated mycoviruses also have potential to be used to control fungal disease [34]. With the exception of SsHADV1 [35, 36], mycoviruses lack extracellular phases in their life cycles and are not known to have transmission vectors; they are transmitted vertically via propagation and horizontally via hyphal anastomosis. To date, only very few mycoviruses that infect ascomycetous fungi were reported to be transmitted via sexual spores, most mycoviruses are transmitted via asexual spores [37, 38]. For fungi that do not produce asexual spores, such as *Sclerotinia sclerotiorum* and *Rhizoctonia solani*, mycoviruses are transmitted and spread via hyphal anastomosis. However, the spread of mycoviruses is limited among vegetatively incompatible individuals, and this limitation is regarded as one of the critical factors responsible for reducing the efficacy of hypovirulence-associated mycoviruses in controlling fungal diseases [12, 22–24, 39].

The family *Reoviridae* consists of two subfamilies (*Spinareovirinae* and *Sedoreovirinae*) based on morphology of virus particle surface and is divided into 15 genera [40]. Reoviruses have diverse host ranges and could infect vertebrates, plants, insects, and fungi. Three members of the genus *Mycoreovirus* have been isolated and characterizated from two filamentous fungi of *C*. *parasitica* (the natural host of CpMYRV1 and CpMYRV2) and *Rosellinia necatrix* (the natural host of RnMYRV3) [41, 42]. CpMYRV1 and RnMYRV3 confer hypovirulence on their hosts. Recent research revealed that CpMYRV1 infection markedly induced the expression of key RNA silencing genes in *C*. *parasitica*, and strongly suppressed replication of a heterologous virus, Rosellinia necatrix victorivirus 1, via an antiviral RNA silencing mechanism [8]. Moreover, RNA silencing played a significant role in the events of genome rearrangements of CpMYRV1 in *C*. *parasitica* [43].

*S*. *sclerotiorum* is an economically important necrotrophic fungal pathogen with a worldwide distribution and infects more than 400 dicotyledonous host species [44]. Various mycoviruses have been identified and characterized from *S*. *sclerotiorum* [34]. Although *S*. *sclerotiorum* has a complex vegetative compatibility groups [45], mycoviruses are widely spread in different vegetative groups, and co-infection events in a single strain were found with high frequency [34]. These phenomena suggested that mycoviruses are likely to overcome the limitation of transmission caused by vegetative incompatibility via some unknown mechanisms. In this study, we report a hypovirulence-associated mycoreovirus that infects *S*. *sclerotiorum* and that could suppress host vegetative incompatibility reaction, and facilitate transmission of phylogenetically unrelated mycoviruses.

## Results

### SsMYRV4 confers hypovirulence on *S*. *sclerotiorum*

**Fig 1:**
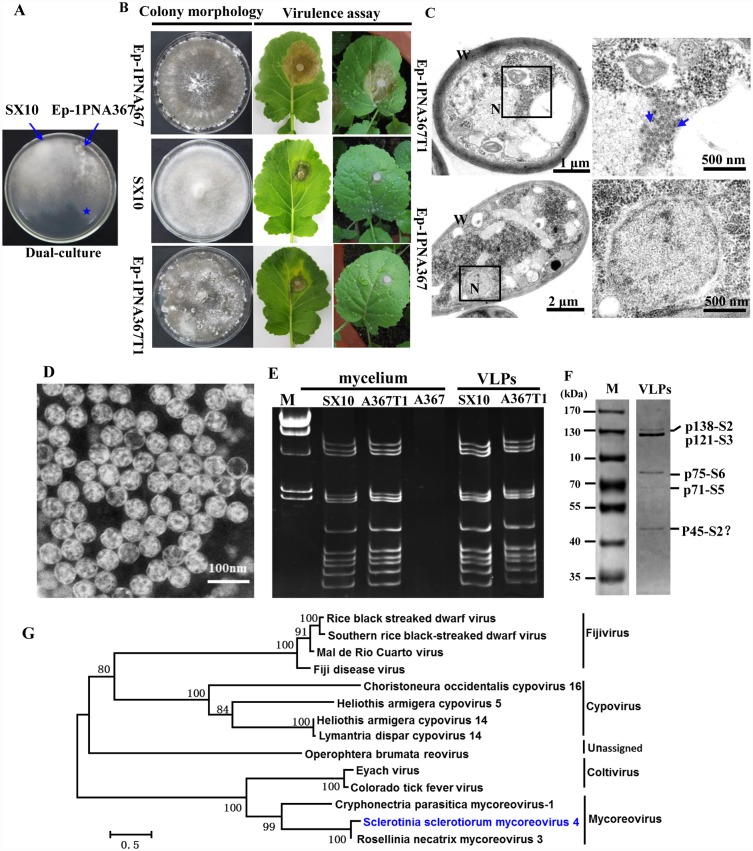
SsMYRV4 confers hypovirulence on *S*. *sclerotiorum*. (A) Hypovirulent strain SX10 was dual-cultured with a virulent strain (such as Ep-1PNA367R). When the hyphae of the two strains contacted each other for 2 days, a mycelial plug potentially containing a newly transmitted mycovirus was picked up from the colony margin (marked with a blue star) of virulent strain (a site farthest from the hypovirulent strain). (B) Biological characteristics of strains Ep-1PNA367, SX10 and Ep-1PNA367T1 (derived from a dual culture of strains Ep-1PNA367 and SX10; as shown in A). All strains were cultured on PDA plates for 10 days prior to photography. Pathogenicity assay of *S*. *sclerotiorum* strains were carried out on detached leaves (left panels) and whole rapeseed plants (right panels). Photos and data were taken at 72 hour post inoculation. (C) Ultrastructure of fungal cells of strains Ep-1PNA367 and Ep-1PNA367T1 as observed under TEM. The two strains were cultured for 4 days and then examined by TEM. The right panel is an enlargement of nuclear area that is indicated by boxes in left panels. VLPs in the cell (right panel) were marked with blue arrows. N = Nucleus, W = cell wall. (D) TEM images of negatively stained SsMYRV4 particles. (E) PAGE analysis on 5% polyacrylamide gel of dsRNA directly extracted from mycelia of strains SX10, Ep-1PNA367T1 (A367T1), Ep-1PNA367 (A367), or extracted from purified virus particles from SX10, Ep-1PNA367T1. Lane M, λ Hind III-digested DNA used as size markers. All dsRNA samples were treated with DNase I and S1 nucleases prior to electrophoresis. (F) SDS-PAGE analysis of purified SsMYRV4 particles. Samples collected from sucrose gradient fractions were subjected to SDS-PAGE analysis on a 12% polyacrylamide gel. The sizes of the Coomassie blue-stained proteins were estimated by comparison with protein size markers (lane M). (G) Phylogenetic analysis of SsMYRV4. A neighbor-joining phylogenetic tree was constructed based on the complete amino acid sequences of viral RdRp. The virus name (Sclerotinia sclerotiorum mycoreovirus 4) was printed in blue color and pertinent information on other reoviruses selected for phylogenetic analysis is shown in S3 Table. Bootstrap values (%) obtained with 2000 replicates are indicated on the branches, and branch lengths correspond to genetic distance; scale bar at lower left corresponds to genetic distance.

Strain Ep-1PNA367 (see Table 1 for a list of all strains used in this study) is a typical *S*. *sclerotiorum* strain with strong virulence on rapeseed plant. Compared with strain Ep-1PNA367, strain SX10 showed abnormal colony morphology with abundant aerial mycelium, less pigment and reduced mycelial growth rate (Fig 1B). Thus strain SX10 exhibits hypovirulence traits similar to those previously reported in *S*. *sclerotiorum* strains infected with diverse RNA or DNA mycoviruses [27]. We subsequently extracted dsRNA and DNA elements from strain SX10. No extra DNA elements were obtained by relevant DNA extraction methods [49]. The results of dsRNA extraction suggested that strain SX10 harbors twelve dsRNA segments, which were designated segment 1 (S1) to segment 12 (S12) in order of increasing electrophoretic mobility on 5% polyacrylamide gels (Fig 1E). We hereafter confirmed that the extracted twelve dsRNA segments comprise the genome of a novel mycoreovirus and tentatively designated this mycoreovirus as Sclerotinia sclerotiorum mycoreovirus 4 (SsMYRV4), following the convention used in naming previously reported three mycoreoviruses in *C*. *parasitica* (CpMYRV1 and CpMYRV2) and *R*. *necatrix* (RnMYRV3). To define whether SsMYRV4 is responsible for phenotypic traits of strain SX10 as compared with strain Ep-1PNA367R, SsMYRV4 was successfully transferred from strain SX10 to strain Ep-1PNA367R via horizontal transmission (Fig 1A), and confirmed by dsRNA extraction and subculturing on PDA medium containing hygromycin. To avoid the influence of T-DNA insertional mutants, SsMYRV4 was further horizontally transferred from strain Ep-1PNA367R to strain Ep-1PNA367. The newly SsMYRV4-infected isolate of Ep-1PNA367 was named Ep-1PNA367T1. The results of dsRNA extraction suggested that Ep-1PNA367T1 harbors 12 dsRNA segments, which were similar to those from strain SX10 (Fig 1E). The assay of biological properties including growth rate, yield of sclerotia and virulence revealed that Ep-1PNA367T1 exhibits hypovirulence and its associated phenotypes (Fig 1B and S1 Fig). Moreover, introduction of purified SsMYRV4 particles into strain Ep-1PNA367 resulted in reductions in virulence and mycelial growth (S2 Fig). Thus, combination of virus transfection and transmission results suggested that SsMYRV4 is associated with debilitation symptoms of *S*. *sclerotiorum*.

**Table 1:** List of *S*. *sclerotiorum* strains used in the study.

Strain	Mycoviruses content*	Background strain	Hygromycin	Compatible/Incompatible with Ep-1PNA367	Reference
Ep-1PNA367	-	Ep-1PNA367	S^%^	Compatible	[46]
Ep-1PNA367R	-	Ep-1PNA367	R	Compatible	This study
1980	-	1980	S	Incompatible	This study
1980R	-	1980	R	Incompatible	This study
RL26	-	RL26	S	Incompatible	This study
DT47-39	-	DT47-39	S	Incompatible	This study
RL19	-	RL19	S	Incompatible	This study
Ep-1PN	SsDRV, SsRV-L	Ep-1PNA367	S	Compatible	[46, 47]
HC025	SsMV1	HC025	S	Compatible	[48]
SX10	SsMYRV4	SX10	S	Compatible	This study
Ep-1PNA367T1^#^	SsMYRV4	Ep-1PNA367	S	Compatible	This study
Ep-1PNA367PT1^$^	SsMYRV4	Ep-1PNA367	S	Compatible	This study
Ep-A367T1	SsMYRV4, SsDRV, SsRV-L	Ep-1PNA367	S	Compatible	This study
HC-A367T1	SsMYRV4, SsMV1	Ep-1PNA367	S	Compatible	This study
EA367T1-1980	SsDRV, SsRV-L	1980	S	Incompatible	This study
EA367T1-1980R	SsDRV, SsRV-L	1980	R	Incompatible	This study
HCA367T1-1980	SsMV1	1980	S	Incompatible	This study
EA367T1-RL19	SsDRV, SsRV-L	RL19	S	Incompatible	This study
HCA367T1-RL19	SsMV1	RL19	S	Incompatible	This study

*The full names of mycoviruses in the list: SsDRV, Sclerotinia sclerotiorum debilitation associated RNA virus; SsRV-L, Sclerotinia sclerotiorum RNA virus L; SsMV1, Sclerotinia sclerotiorum mitovirus 1; SsMYRV4, Sclerotinia sclerotiorum mycoreovirus 4.

- No mycovirus was detected with dsRNA extraction method

^#^ Strain Ep-1PNA367T1 was derived from a dual culture of strains SX10 and Ep-1PNA367.

^$^ Strain Ep-1PNA367PT1 was a transfectant of strain Ep-1PNA367 with SsMYRV4 purified particles.

^%^ R donates that the strain is resistant to hygromycin (30 μg/ml), and S indicates that the strain is susceptible to hygormycin

The hyphal ultrastructures of strains Ep-1PNA367T1 and Ep-1PNA367 were examined by transmission electron microscope (TEM). Compared to strain Ep-1PNA367, most of the nucleoli of strain Ep-1PNA367T1 were disintegrated with dispersed chromatin, and some mitochondria were destroyed or swelled (Fig 1C). More interestingly, some spherical virus-like particles (VLPs) with sizes of 65~70 nm in diameter were localized near the nuclear membrane area (Fig 1C), which is reminiscent of viral factory-like areas encapsulated with double membrane structure, as previously reported in virus-infected plants [50]. Similar structures were not observed in strain Ep-1PNA367. Purified virus particles of SsMYRV4 were successfully obtained from mycelia of strain Ep-1PNA367T1, and the 60~70 nm double-shelled spherical particles (Fig 1D) were similar in size to VLPs observed in hyphal thin sections of Ep-1PNA367T1. The 12 dsRNA segments (SsMYRV4 genome) released from purified virus particles were consistent in size with that directly isolated from mycelium of Ep-1PNA367T1 and SX10 (Fig 1E).

The 12 dsRNA segments of SsMYRV4 were fractioned and purified, and then subjected to cDNA cloning. A summary of the cDNA sequence information of the 12 dsRNA segments is listed in S1 Table. The size of full-length cDNAs of the 12 dsRNA segments ranges from 1000 bp to 4143 bp. A single open reading frame (ORF) was detected on the positive strand of each dsRNA segment, and a total of 12 putative proteins (VP1 to VP12) were predicted. In addition, the terminal sequences (5’-ACAAUUU—UGCAGAC-3’) are conserved in all 12 dsRNA segments (S3 Fig). A BLASTp search of the NCBI protein database and multiple sequence alignments were performed with the 12 putative proteins encoded by S1 to S12. The protein VP1 encoded by S1 has the typical I-VII motifs of RNA-dependent RNA polymerase (RdRp) (S4 Fig), and shares 91% identity with Rosellinia necatrix mycoreovirus 3 (RnMYRV3) of the genus *Mycoreovirus* in family *Reoviridae* (S1 Table). Phylogenetic analysis of the RdRp sequence further confirmed that SsMYRV4 is closely related to RnMYRV3 (S2 Table and Fig 1G). Other putative proteins encoded by S2 to S12 also share similarities to the corresponding 11 proteins encoded by RnMYRV3 with 40% to 92% identity (S1 Table).

Five distinct protein bands (p140, p120, p75, p70, and p45) were obtained when purified SsMYRV4 particles were subjected to SDS-PAGE on a 12% polyacrylamide gel (Fig 1F). These five protein bands were individually subjected to polypeptide mass fingerprint (PMF) analysis. More detailed information on peptide fragment analysis was provided in S3 Table. The above results revealed that VP2 and VP3 represent the major structural proteins of SsMYRV4 virions, whereas VP5 and VP6 comprise minor structural proteins of SsMYRV4 virions.

### SsMYRV4 suppresses non-self recognition of *S*. *sclerotiorum*

**Fig 2:**
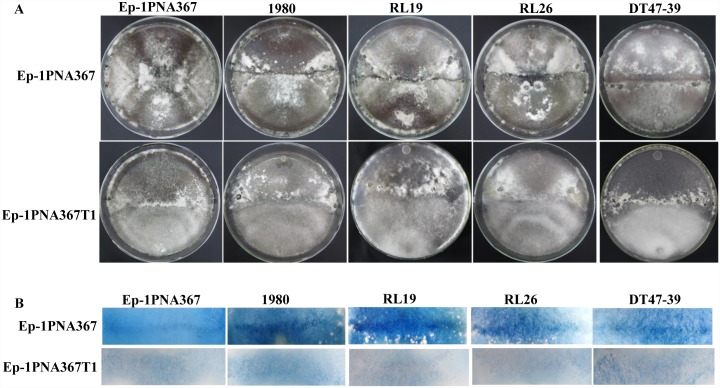
SsMYRV4-mediated suppression of vegetative incompatibility reaction and PCD response. (A) Strains Ep-1PNA367 (SsMYRV4-free) and Ep-1PNA367T1 (SsMYRV4-infected) were each dual-cultured with strains Ep-1PNA367, 1980, RL19, RL26 and DT47-39 on PDA amended with 75 μl/L of McCormick’s red food coloring. A narrow intense-black-pigmentation zone was easily observed in the interface region of Ep-1PNA367 and other referenced strains, but such a black-pigmented zone was not detected when Ep-1PNA367 was replaced with Ep-1PNA367T1. The two strains were individually dual-cultured for 10 days. (B) Evans blue staining to check the PCD response. PCD response was detected at the interface regions after the hyphae of individuals *S*. *sclerotiorum* strains contacted each other for 3 hours.

In filamentous fungi, non-self recognition causes death of the interacting hyphae via a PCD mechanism. When two hyphae from vegetatively incompatible individuals contact each other, this non-self recognition-mediated PCD often manifests as a macroscopic phenotype of slow growth and black-pigmented bands bordering each side of the two contacting fungal isolates and a microscopic phenotype of hyphal compartmentation and cell death [20]. When Ep-1PNA367 was dual-cultured with four other *S*. *sclerotiorum* strains (1980, RL19, RL26, and DT47-39) on a PDA plate, a clear intense black-pigmented zone (an evident antagonism line) appeared at the interface region, which revealed that a strong incompatibility reaction exists between Ep-1PNA367 and each of the other four strains of *S*. *sclerotiorum* (Fig 2A). However, Ep-1PNA367T1 carrying SsMYRV4 did not exhibit vegetative incompatibility reactions with these four strains (Fig 2A). Previous reports revealed that vegetative incompatibility reactions were usually triggered by localized PCD [20]. Thus, the Evans blue staining assay was applied to detect PCD at the interface region of two *S*. *sclerotiorum* strains, as shown in Fig 2B. The results suggested that cell death occurred at the interface region of strain Ep-1PNA367 and the other four strains, whereas cell death was markedly decreased when Ep-PNA367 was replaced with Ep-1PNA367T1. Based on these results, we concluded that SsMYRV4 could suppress non-self recognition-mediated PCD in *S*. *sclerotiorum*.

### SsMYRV4 inhibits expression of heterotrimeric G protein subunit genes in *S*. *sclerotiorum*

**Fig 3:**
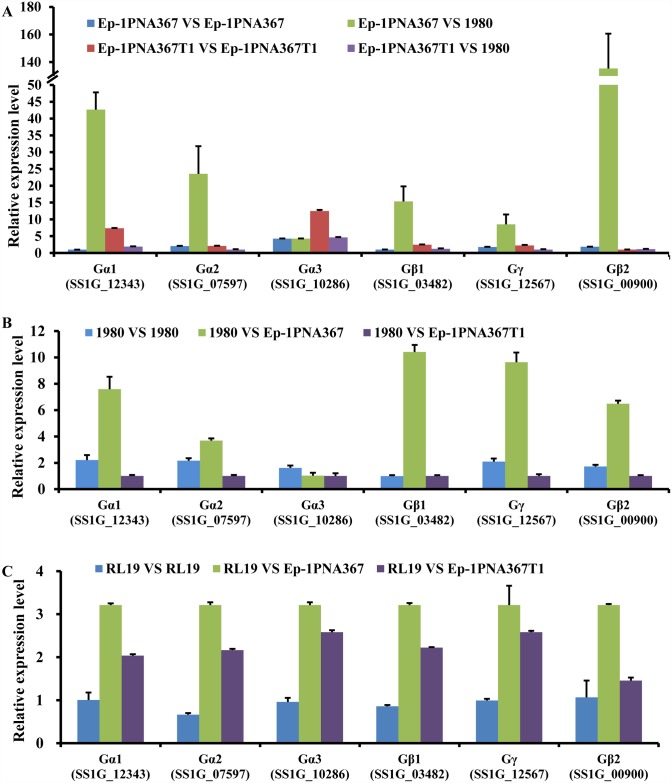
SsMYRV4-mediated suppression of G protein subunit genes expression in *S*. *sclerotiorum*. (A) qRT-PCR analysis of expression level of G protein subunit genes in mycelium from strains Ep-1PNA367 (Ep-1PNA367 VS Ep-1PNA367), Ep-1PNA367T1 (Ep-1PNA367T1 VS Ep-1PNA367T1), and mycelium from Ep-1PNA367 dual-cultured with strain 1980 (Ep-1PNA367 VS 1980), Ep-1PNA367T1 dual-cultured with 1980 (Ep-1PNA367T1 VS 1980). (B) qRT-PCR analysis of expression level of G protein subunit genes in mycelium from strain 1980 (1980 VS 1980), strain 1980 dual-cultured with Ep-1PNA367T (1980 VS Ep-1PNA367) or Ep-1PNA367T1 (1980 VS Ep-1PNA367T1). (C) qRT-PCR analysis of expression level of G protein subunit genes in mycelium from strain RL19 (RL19 VS RL19), strain RL19 dual-cultured with Ep-1PNA367T (RL19 VS Ep-1PNA367) or Ep-1PNA367T1 (RL19 VS Ep-1PNA367T1). The mycelium of *S*. *sclerotiorum* strains was collected at 5 hours post-contact with vegetatively incompatible or compatible strains.

Heterotrimeric G proteins as multifunctional signaling proteins are essential for growth, asexual and sexual development, and vegetative incompatibility in fungi [17]. Heterotrimeric G protein contains three subunits, namely Gα, Gβ and Gγ, and three homologues of Gα (SS1G_07597, SS1G_12343, and SS1G_10286), one Gβ (SS1G_03482) and one Gγ (SS1G_12567) have been identified in *S*. *sclerotiorum* [17]. In addition, we found that SS1G_00900 was similar to SS1G_03482 encoding guanine nucleotide-binding protein β subunit. With exception of the Gα subunit gene (SS1G_10286), the expression of the other five G protein subunit genes were significantly up-regulated when hyphae from two incompatible individuals (1980 and Ep-1PNA367) contacted each other (Fig 3A), but down-regulated in strain Ep-1PNA367T1 due to SsMYRV4 infection (Fig 3A). Furthermore, the expression of G protein subunit genes were also suppressed in SsMYRV4-free strain 1980 or RL19 when the hyphae of strain 1980 or RL19 came in contact with SsMYRV4-infected vegetatively incompatible strain Ep-1PNA367T1 (1980 VS Ep-1PNA367T1 or RL19 VS Ep-1PN367T1) (Fig 3B and 3C). Therefore, G protein subunit genes involved in the process of fungal non-self recognition in *S*. *sclerotiorum*, and SsMYRV4-infection can suppress the expression of G protein subunit genes in two different incompatible pairings (Fig 3B and 3C).

### SsMYRV4 suppresses expression of HET-domain related genes in *S*. *sclerotiorum*

**Fig 4:**
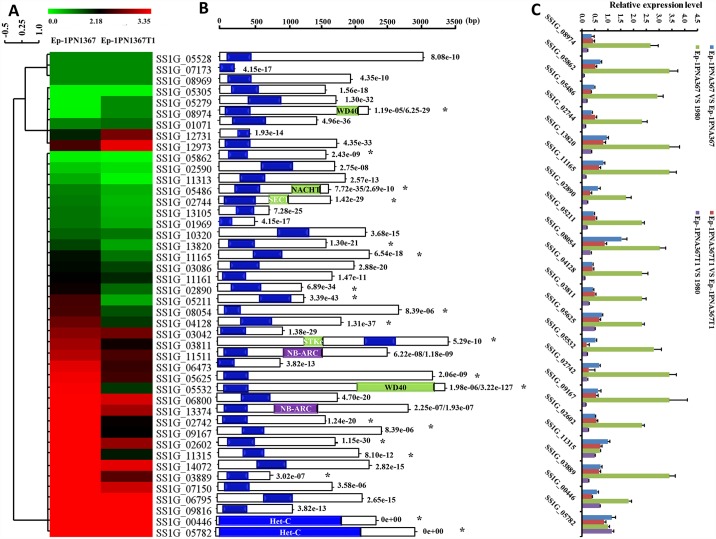
Transcriptional analysis and organization of the candidate *het* genes that encoded proteins containing HET conserved domains in *S*. *sclerotiorum*. (A). Expression cluster analysis of *het* genes in strains Ep-1PNA367 and Ep-1PNA367T1. The relative expression of *het* genes was analyzed based on the threshold of RPKM value. Red means high expression level and green indicates low expression level. The expression profile was analyzed based on RNA-seq data (GEO accession number GSE94575) (B) Organization of *het* genes in *S*. *sclerotiorum*. The HET conserved domains were shown with blue frame. The information (conserved domain, e-value and gene length) of *het* genes was obtained from NCBI database. The genes selected for qRT-PCR analysis were marked with a black star. (C) qRT-PCR analysis of expression level of twenty *het* genes during hyphal fusion progress of two vegetatively incompatible or compatible strains. Total RNA was extracted from mycelium of strains Ep-1PNA367 (Ep-1PNA367 VS Ep-1PNA367), Ep-1PNA367T1 (Ep-1PNA367T1 VS Ep-1PNA367T1), and mycelium from Ep-1PNA367 dual-cultured with strain 1980 (Ep-1PNA367 VS 1980), Ep-1PNA367T1 dual-cultured with 1980 (Ep-1PNA367T1 VS 1980)

HET-domain related genes (*het* genes or *vic* genes) that encode proteins containing conserved HET domains (*het* genes) are often thought to be involved in heterokaryon (vegetative) incompatibility [14, 51]. Forty-four genes (*het* genes) encoded proteins were predicted to contain a HET domain in the genome of *S*. *sclerotiorum* (Fig 4B), thirty-five of which were down-regulated as the result of SsMYRV4 infection based on RNA-seq data (Gene Expression Omnibus, GEO accession number GSE94575) (Fig 4A). The expression level of twenty *het* genes with high similarity to previously reported *het* genes was further monitored during hyphal contact progress between strain Ep-1PNA367 (or strain Ep-1PNA367T1) and strain 1980 by qRT-PCR analysis. The results suggested that the expression of eighteen *het* genes was significantly induced in the SsMYRV4-free strain (Ep-1PNA367 and 1980), while eighteen of these twenty *het* genes were significantly down-regulated in SsMYRV4-infected strains, and the remaining two *het* genes were slightly up-regulated. (Fig 4C). Therefore, we concluded that *het* genes were significantly up-regulated during the process of fungal non-self recognition, and SsMYRV4-infection can suppress the expression of *het* genes in *S*. *sclerotiorum*.

### SsMYRV4 inhibits ROS production in *S*. *sclerotiorum*

**Fig 5:**
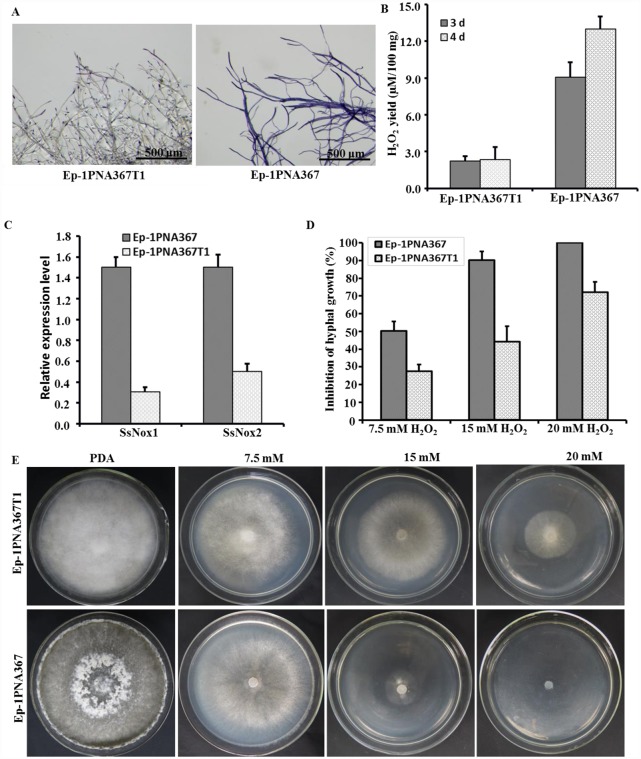
SsMYRV4-mediated inhibition of ROS production in hyphae of *S*. *sclerotiorum*. (A) NBT staining of hyphae of strains Ep-1PNA367 and Ep-1PNA367T1. Strains were cultured on CM-PDA plates for 2 days and stained with 0.5% NBT in phosphate buffer solution (1 mol/L) for 1 hour under dark conditions, and then the reaction was terminated with anhydrous ethanol. (B) Quantitative detection of H_2_O_2_ level in hyphae of strains Ep-1PNA367 and Ep-1PNA367T1. Samples were harvested and H_2_O_2_ level was measured at 3 and 4 dpi. (C) Relative expression of two ROS-associated genes, *Ssnox1* and *Ssonx2*, in Ep-1PNA367 and Ep-1PNA367T1 via Real-time PCR. Total RNA was extracted from mycelium cultured for 3 days and used for qRT-PCR analysis. Comparison of hyphal growth inhibition (D) and colony morphology (E) of strains Ep-1PNA367 and Ep-1PNA367T1 grown on PDA containing 7.5 mM, 15 mM or 20 mM H_2_O_2_.

Reactive oxygen species (ROS) is associated with PCD and plays a critical role in fungal vegetative incompatibility reaction [52, 53]. The cellular ROS level was evaluated in SsMYRV4-infected and SsMYRV4-free strains. The nitro blue tetrazolium (NBT) dye results showed that strain Ep-1PNA367T1 produced less ROS than strain Ep-1PNA367 at the vegetative stage (Fig 5A). Quantitative determination of cellular H_2_O_2_ concentration indicated that H_2_O_2_ level in strain Ep-1PNA367T1 was significantly reduced compared to that in strain Ep-1PNA367 (Fig 5B). Two *S*. *sclerotiorum* NADPH oxidases genes (*Ssnox1* and *Ssnox2*) are known to be involved in ROS production [54]; these were assayed in SsMYRV4-infected and free strains. Consistent with ROS staining and H_2_O_2_ determination, the expressions of both *Ssnox1* and *Ssnox2* were significantly down regulated 3 to 5 times in strain Ep-1PNA367T1 (Fig 5C). These results suggested that SsMYRV4 infection could play a functional role in inhibition of ROS production via down-regulation of ROS-associated genes in *S*. *sclerotiorum*.

Ep-1PNA367 and Ep-1PNA367T1 were cultured on PDA medium amended with different concentrations of H_2_O_2_. The growth of strain Ep-1PNA367 was significantly suppressed on PDA containing 15 mM H_2_O_2_, and completely suppressed in the presence of 20 mM H_2_O_2_. However, the growth of strain Ep-1PNA367T1 was not significantly impacted under the same experimental conditions (Fig 5D and 5E). These findings suggested that resistance of Ep-1PNA367T1 to H_2_O_2_ correlated with the presence of SsMYRV4.

### Infection with SsMYRV4 facilitates horizontal transmission of heterologous mycoviruses between different vegetative compatibility groups

**Fig 6:**
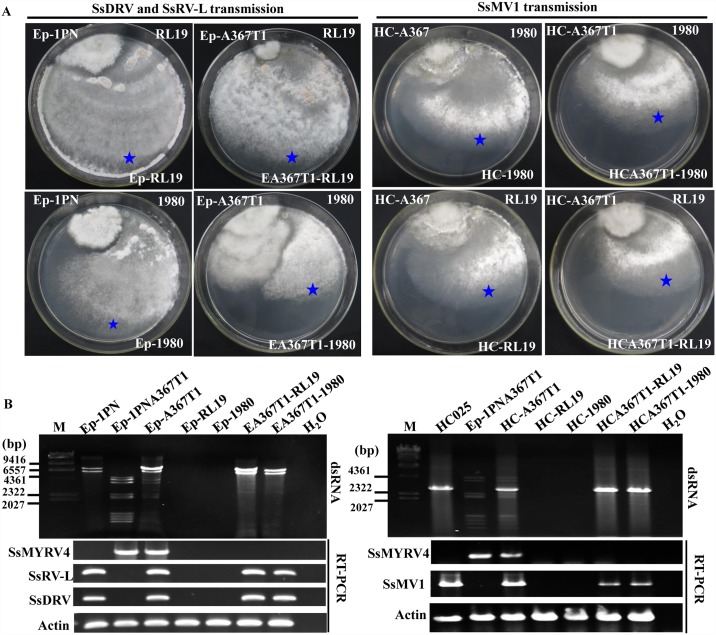
SsMYRV4-mediated enhancement of horizontal transmission of unrelated mycoviruses among *S*. *sclerotiorum* individuals belonging to different VCGs. (A) Horizontal transmission of three mycoviruses SsDRV, SsRVL and SsMV1 using a dual-culture method on PDA plates (15-cm in diameter). SsDRV and SsRVL co-infect hypovirulent strain Ep-1PN, whereas SsMV1 naturally infects hypovirulent strain HC025. SsMV1 was horizontally transmitted into strain Ep-1PNA367 under laboratory conditions and the newly obtained SsMV1-infected strain was named HC-A367. Ep-A367T1 harbors three mycoviruses (SsDRV, SsRVL, and SsMYRV4). HC-A367T1 carries two mycoviruses (SsMV1 and SsMYRV4). After the mycelia of two different strains (a hypovirulent strain and a virulent strain) contact each other for 7 days, the new isolates (indicated by blue stars) were picked up from colony margin of the virulent strain, and the designations of newly obtained isolates are shown at the bottom of each plate. (B) Mycovirus content was assessed by dsRNA extraction and RT-PCR amplification. Primers SsMYRV4F and SsMYRV4R, SsDRV-F and SsDRV-R, SsRVLF and SsRVLR, and SsMV1-F and SsMV1-R (S5 Table) were used for detection of SsMYRV4, SsDRV, SsRVL, and SsMV1, respectively. The *actin* gene served as an internal control; sizes of molecular mass standards (M) are indicated to the left of each panel.

Fungal vegetative incompatibility reaction usually reduces the efficiency of mycovirus horizontal transmission, and is regarded as a key barrier to limit exploitation of mycoviruses as biological control agents. Since SsMYRV4 can suppress the non-self recognition system in *S*. *sclerotiorum*, we further tested whether SsMYRV4 could be horizontally transmitted with higher frequency between different vegetative compatibility groups (VCGs). The results of horizontal transmission experiments revealed that it is difficult for SsMYRV4 to be horizontally transmitted among *S*. *sclerotiorum* individuals regardless of the VCGs of the contacting strains (Fig 6B). Following mycelial contact between strains Ep-1PNA367T1 (infected with SsMYRV4) and Ep-1PN (co-infected with SsDRV and SsRV-L), or strains Ep-1PNA367T1 and HC-A367 (same genetic background as Ep-1PNA367, but infected by SsMV1), three mycoviruses were successfully transmitted to strain Ep-1PNA367T1 (infected with SsMYRV4), and the newly mycovirus-infected isolates were designated as Ep-A367T1 (co-infected with SsDRV, SsRV-L, and SsMYRV4) and HC-A367T1 (co-infected with SsMV1 and SsMYRV4). Four mycovirus-infected strains (Ep-1PN, HC-A367, HC-A367T1 and Ep-A367T1, see Table 1) were, respectively, dual-cultured with two mycovirus-free strains 1980 and RL19 on PDA plates. Mycelial agar plugs were picked up from colony margins of strain 1980 and RL19 (at sites farthest from strains Ep-1PN, HC-A367, HC-A367T1 and Ep-A367T1). All newly obtained isolates were sub-cultured for three rounds, and then used for detecting mycovirus contents by analyzing extracted dsRNA via agarose gel electrophoresis and RT-PCR. The results suggested that subcultures of strains 1980 and RL19 dual-cultured with SsMYRV4-infected strains (Ep-A367T1 and HC-A367T1) harbored SsDRV and SsRV-L, or SsMV1, whereas these three mycoviruses were not detected in subcultures of strains 1980 and RL19 dual-cultured with SsMYRV4-free strains Ep-1PN and HC-A367 (Fig 6B). The other vegetatively incompatible strains (RL26, RL19, and DT47-39 as recipient strains) were dual-cultured with Ep-1PN and Ep-A367T1 (as donor strains), and the results indicated that SsDRV and SsRV-L could horizontally be transmitted from donor SsMYRV4-infected strains to recipient strains, but failed to be transmitted in the absence of SsMYRV4 in the donor strain (S4 Table). These results indicated that infection with SsMYRV4 could serve as a bridge to facilitate horizontal transmission of heterologous viruses between different VCGs in *S*. *sclerotiorum*.

### SsMYRV4 infection benefits spread of heterologous mycoviruses on rapeseed plant

**Fig 7:**
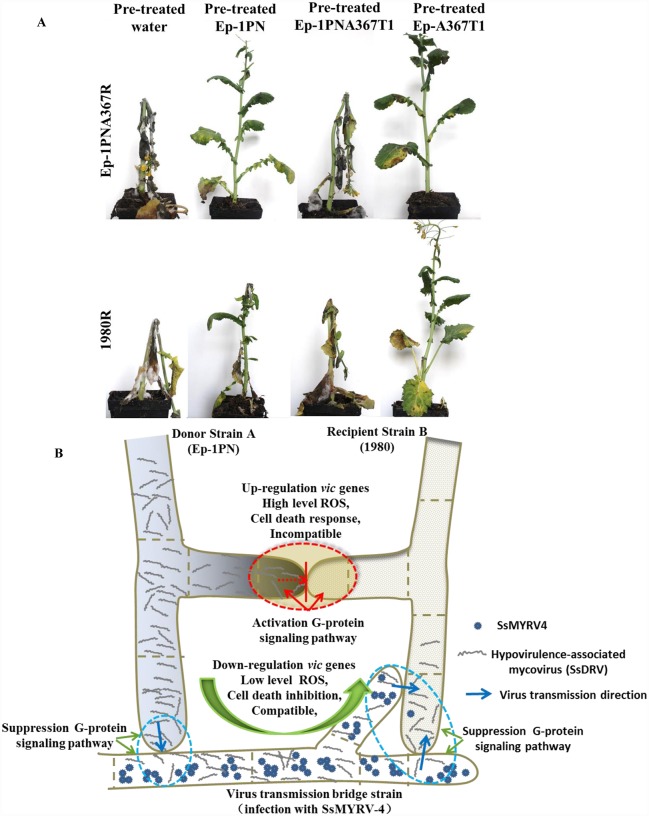
SsMYRV4-mediated enhancement of horizontal transmission between different VCGs effectively prevents and controls Sclerotinia diseases. (A) SsMYRV4-mediated horizontal transmission of SsDRV controls Sclerotinia diseases on plants of *B*. *napus*. Plant leaveas were pre-treated with mycelial suspension (2 g/40ml) of Ep-1PN (infected with SsDRV), Ep-1PNA367T1 (SsMYRV4) and Ep-A367T1 (co-infected with SsDRV and SsMYRV4) for 3 days, and then treated with mycelial suspension of Ep-1PNA367R and 1980R. Photographs were taken at 5 days posted inoculation of Ep-1PNA367R and 1980R. (B) A model for SsMYRV4-mediated horizontal transmission between different VCGs. Strains 1980, Ep-1PN and the bridge donor strain belong to different VCGs. Vegetative incompatibility results in death of the interacting hyphae via PCD (red dotted line area), and in blocking virus horizontal transmission.

SsDRV confers hypovirulence on *S*. *sclerotiorum* [46], but SsDRV could not be horizontally transmitted from strain Ep-1PN to strain 1980 via hyphal contact due to strain Ep-1PN and strain 1980 belong to different VCGs. The SsDRV-infected strain Ep-1PN could not be effectively employed for control of diseases caused by virulent strain 1980 on living plants (Fig 7A). Therefore, we further evaluated whether SsMYRV4 could promote SsDRV transmission in living plants of *B*. *napus* or *N*. *benthamiana*. Mycelial suspensions of strains Ep-1PN (co-infected with SsDRV and SsRV-L), Ep-1PNA367T1 (infected with SsMYRV4) and Ep-A367T1 (co-infected with SsDRV, SsRV-L and SsMYRV4) were sprayed on *N*. *benthamiana* or *B*. *napus* leaves, and then virulent strain 1980R or Ep-1PNA367R was sprayed on the pre-treated plants. Although mock (treatment with water) and Ep-1PNA367T1 pre-treated plants were essentially killed and dead at 5 days-post-inoculation (dpi), Ep-A367T1 pre-treated plants were still alive (Fig 7A and S5A Fig). Furthermore, 1980R isolates from diseased stalk were further cultured on hygromycin-containing PDA medium. The results showed that new 1980R isolates from Ep-A367T1 pre-treated plants carried SsDRV and their colony morphology showed disease phenotypes (S5B and S5C Fig). These results suggested that infection of *S*. *sclerotiorum* with SsMYRV4 could potentially be exploited as a bridge donor for heterologous hypovirulence-associated mycoviruses to be spread under natural conditions for control of fungal diseases (Fig 7B).

## Discussion

In this study, we identified a fungal reovirus, SsMYRV4, from a hypovirulent strain SX10 of *S*. *sclerotiorum*. SsMYRV4 suppressed non-self recognition reaction by down-regulating expression of *vic* (or *het*) genes. The determinants of vegetative incompatibility for mycoviruses horizontal transmission of mycoviruses has been thoroughly researched in *C*. *parasitica*-CHV1 interaction system. Six *vic* loci with two alleles at each locus have been so far defined in *C*. *parasitica* [16], and disruption of genes *vic1*, *vic2*, *vic3*, *vic6*, or *vic7* resulted in increasing CHV1 horizontal transmission [23]. *S*. *sclerotiorum* has a complex vegetative incompatibility system [45, 55] and has diverse candidate *het* genes. Screening the published genome data [56] and our RNA-seq data (GEO accession number GSE94575) of *S*. *sclerotiorum*, forty-four genes encoded proteins were shown to contain conserved HET domains. Moreover, five of these forty-four genes contain other conserved sequences typical of non-self recognition domains (WD40, NB-ARC, NACHT). In addition to HET domains, these genes share high sequence identities with previously reported *vic* genes in *N*. *crassa* and *P*. *anserina* [14, 57]. However, the mechanisms underlying SsMYRV4-mediated down-regulation of *het* genes remain to be unraveled.

Although some hypovirulence-associated mycoviruses have been characterized as potential biological control agents against fungal plant diseases, the complicated vegetative incompatibility systems in filamentous fungi have hampered implementation of virocontrol strategies to control fungal disease under natural conditions [58]. To break or attenuate the obstacle of vegetative incompatibility that limits mycovirus horizontal transmission, several possible strategies have been previously employed. First, an increasing number of novel mycoviruses with strong infectivity were subjected to screening assays in plant pathogenic fungi. For example, two *Heterobasidion* partitiviruses (HetRV3-ec1 and HetRV4-pa1) can be readily transmitted among vegetatively incompatible individuals [59]. SsHADV-1 particles could directly infect hyphae of its host [35]. Furthermore, SsPV1 provides another example for a mycovirus that can be transmitted interspecifically among *Sclerotinia* species via hyphal contact [60]. Second, the vegetative incompatibility reaction could be attenuated by amending the growth medium with certain chemicals, e.g., zinc chloride treatment was found to suppress vegetative incompatibility reaction in *R*. *necatrix* and to benefit mycovirus horizontal transmission [61]. Third, disruption of *vic* genes resulted in enhancing mycovirus transmission. In *C*. *parasitica*, eleven genes associated with five of the six *vic* loci have been so far identified and confirmed to alter incompatibility reactions or virus transmission [22, 23]. The super donor strains with systematic disruption of multilocus *vic* genes were able to transmit hypoviruses to strains that were heteroallelic at one or all of the virus-restricting *vic* loci [24]. Our present results suggested that infection with SsMYRV4 could strongly attenuate vegetative incompatibility reaction to facilitate horizontal transmission of heterologous viruses among *S*. *sclerotiorum* individuals. Thus SsMYRV4 could be introduced into a strain of *S*. *sclerotiorum* to create a bridge donor strain for heterologous virus transmission, thus providing a fourth possible strategy to break host vegetative incompatibility and facilitate transmission of hypovirulence-associated mycoviruses for biological control under living plant conditions (Fig 7B).

For successful virus infections in nature in a certain host, replication and transmission are two essential requirements. In host cells, viruses have developed various tactics to avoid non-self recognition and to counteract hosts’ antiviral systems to facilitate their own reproduction and spread. Viruses may mimic host genes to survive in host cells [62, 63]. Viruses may utilize their RNA secondary structural elements at the 5’ untranslated regions to escape host non-self recognition [6]. Viruses also can encode RNA-silencing suppressors to inhibit hosts’ antiviral RNAi pathways [64]. However, somatic fusion which is limited by non-self recognition is a key pathway for horizontal transmission of viruses that lack extracellular stages in their life cycles and those for which transmission vectors are not known. Our findings suggest that SsMYRV4-mediated suppression of non-self recognition among host individuals is one strategy for enhancement of virus spread.

The suppression of non-self recognition is likely to exist widely in nature. Previously, Cryphonectria hypovirus 1 (CHV1) was introduced into *C*. *cubensis* and conferred hypovirulence to its new host. Interestingly, CHV1 could not be transmitted via asexual reproduction in *C*. *cubensis*, but could be transmitted via hyphal contact between either vegetatively compatible or incompatible *C*. *cubensis* strains [65]. Biella et al [66] found that CHV1 infection could have a significant positive or negative effect on PCD in *C*. *parasitica* based on the specific *vic* loci of tested strains. If *vic3* was heteroallelic, cell death frequency was significantly reduced in the virus-free recipient isolates, thus the marked reduction of PCD might promote virus transmission. However, if two *vic* loci (*vic3*, *vic7*) were different between donor and recipient strains, the transmission efficiency was very low with rare exceptions [39]. Brusini et al [12], on the other hand, found that transmission of CHV1 among vegetatively incompatible strains (with different two or four *vic* loci) of *C*. *parasitica* on chestnut stem (in planta) was observed with high frequency (transmission rate >50%). Transmission of a mycovirus-like double-stranded (ds) RNA element (N10) between vegetatively incompatible strains of *R*. *necatrix* was observed when the N10-infected strain and a virus-free strain were co-inoculated on the roots of an apple tree [67]. These discoveries along with our findings suggested that fungal non-self recognition systems can be weakened under certain conditions.

Plant pathogenic fungi could encounter multiple environmental stresses during successful infection processes since plants usually generate a high level of ROS to prevent pathogen invasion [68]. To confer tolerance to host oxidative burst, some pathogenic fungi (such as *Magnaporthe oryzae*) have evolved to develop a robust anti-oxidant defense system [69]. Previous reports also revealed that endophytic fungi may scavenge damaging ROS generated by plant defense mechanisms in response to environmental stress [70]. SsMYRV4-infected strains exhibited high tolerance to oxidative stress, osmotic pressure and extreme temperatures; whereas colonies of virus-free strains were sensitive to adverse stress (S6 Fig). Thus SsMYRV4 may play an important function to benefit the host fungus in responding to cellular stress via scavenging intracellular ROS and repressing PCD response. Although SsMYRV4 confers hypovirulence on *S*. *sclerotiorum*, SsMYRV4, from an evolutionary point of view, may make effective use of a complex mechanism (inhibition of ROS accumulation level) to enhance its fungal host adaptability to environmental stresses allowing for better survival under natural conditions.

Most reports on mycoviruses have focused on hypovirulence and its potential biocontrol applications [34], but other biological functions of mycoviruses received less attention. Three mycoviruses in *Saccharomyces cerevisiae* encode specific killer toxins and self-protective immunity components. This killer phenomenon was frequently found in other yeasts (*Zygosaccharomyces bailii*, *Hanseniaspora uvarum*) and filamentous fungi (*Ustilago maydis*). These mycovirus-infected strains have remarkable antimycotic activity [71]. Furthermore, superkiller (Ski) proteins of yeast have been confirmed to have functions of anti-RNA virus activities [72]. Although the victorivirus Helminthosporium victoriae virus 190S (HvV190S) lacks viral-encoded antifungal genes, HvV190S-infected strains of two different fungi (*Helminthosporium victoria* and *C*. *parasitica*) exhibited strong antifungal activity [73]. Curvularia thermal tolerance virus (CThTV) isolated from an endophytic fungus can enhance heat tolerance in plants via plant-fungal symbiosis [70]. These examples reveal that mycoviruses have other important beneficial attributes to bestow on their hosts in addition to hypovirulence. In this study, we presented evidence that SsMYRV4 not only enhance *S*. *sclerotiorum* tolerance to stress, but also benefits transmission of heterologous viruses among *S*. *sclerotiorum* individuals belonging to different VCGs. Based on phylogenetic analysis and percent nucleotide sequence identity, RnMYRV3 is closely related to SsMYRV4, yet RnMYRV3 or other mycoviruses have not been reported to benefit transmission of heterologous viruses in ways similar to SsMYRV4. The exclusivity of this biological function to SsMYRV4 remains unexplained. All above studies provide a more comprehensive understanding of the interactions between mycoviruses and fungal hosts. It is noteworthy that, although SsMYRV4 helps horizontal transmission of heterologous viruses with high efficiency, SsMYRV4 is transmitted at low efficiency via horizontal transmission among *S*. *sclerotiorum* individuals. Similarly, RnMYRV3 is transmitted at low efficiency among *R*. *necatrix* isolates [41], but this property of mycoreoviruses transmission remains to be explored.

In summary, we isolated and characterized a new hypovirulence-associated mycoreovirus, SsMYRV4. The purified virus particles contain four structural proteins and TEM revealed the localization of virus particles near the nuclear membrane. SsMYRV4 has the ability to function as a potent inhibitor of fungal non-self recognition via down-regulation of the expression of G protein signaling pathway and eventually inhibiting vegetative incompatibility-mediated PCD in *S*. *sclerotiorum*. SsMYRV4 is capable of breaking vegetative incompatibility barriers and help horizontal transmission of heterologous viruses between different VCGs. Although the mechanism underlying viral-suppression of vegetative incompatibility reaction is still largely unknown, our finding of SsMYRV4-mediated enhancement of transmission and spread of mycoviruses will undoubtedly stimulate future research on virocontrol of *S*. *sclerotiorum* in the field.

## Materials and methods

### Isolates of *S*. *sclerotiorum* and culture methods

*S*. *sclerotiorum* strain SX10 was isolated from a sclerotium obtained from a diseased rapeseed (*B*. *napus*) plant in Ninqiang county, Shanxi province. The hypovirulent strains Ep-1PN and HC025 were infected by two mycoviruses (SsDRV and SsRVL) and a mitovirus (SsMV1) (Table 1), respectively. Strains Ep-1PNA367 and DT47-39 are single-ascospore-isolates derived from strains Ep-1PN and DT-47, respectively. Strains Ep-1PNA367R and 1980R were labeled with a hygromycin-resistance gene (*hph*) (Table 1). Strains Ep-1PNA367, DT47-39, 1980, RL19 and RL26 belong to different VCGs (Table 1). All strains were cultured on PDA medium at 20–22°C, and stored at 4°C. PDA amended with 75 μl/L of McCormick’s red food coloring was used for vegetative compatibility tests [55].

### Intracellular localization, electron microscopy, virus particles purification and transfection

To study intracellular localization of virus particles, ultrathin mycelial sections of mycovirus-infected and mycovirus-free strains were observed under TEM. Briefly, all fungal strains were cultured on PDA plates covered with cellophane membrane (CM-PDA) at 20°C for 6 days. Ultrathin sections were prepared and observed as previously described [60]. At least 10 sections of each sample were examined. Virus particles were purified and visualized using transmission electron microscopy (TEM) as previously described [60]. Viral nucleic acid was extracted from purified virus particles as previously described [60]. The procedure for dsRNA extraction from fungal mycelium was as previously described [47]. All dsRNA samples were further treated with DNase I and S1 nuclease (TaKaRa, Dalian, China), and then fractioned by agarose gel electrophoresis (1% agarose) or polyacrylamide gel electrophoresis (5% polyacrylamide).

Purified virus particles were used for infectivity assays. Protoplasts of strain Ep-1PNA367 was prepared following a previously described method [60]. The protocol for transfection of protoplasts with viral particles (10–20 μg) was as previously described by Xiao *et al*. [60]. Transfection was confirmed by dsRNA extraction and PCR amplification of viral genomic fragments using gene-specific primers (SsMYRV4-F and SsMYRV4-R) listed in S5 Table.

### Genome cloning, sequencing, and sequence analyses

The individual dsRNA segments were separated by gel electrophoresis, and each segment was excised and purified using a gel extraction kit. The purified dsRNA was used to construct libraries by random PCR amplification and for the termini of each dsRNA segment, the phosphorylated 5’-end oligonucleotide (RACE-OLIGO) was ligated to the 3’-terminus of each strand of dsRNA as previously described [47]. All sequences were assembled and aligned with DNAMAN (version 5.2.9, Lynnon Biosoft). The sequence information of previously reported reoviruses referenced in this paper was retrieved from the NCBI GenBank database (http://www.ncbi.nlm.nih.gov/genome/). On the basis of multiple sequence alignments with ClusterX2, neighbor-joining phylogenetic trees were constructed using MEGA version 6.0 programs [74].

### Peptide mass fingerprinting analysis of virus structural proteins

The purified virus particles were boiled for 5 min and then electrophoresed on a 12% polyacrylamide gel using SDS-polyacrylamide gel electrophoresis (SDS-PAGE) for 2 h at 80 V. The gel was then stained with Coomassie brilliant blue R250. Each visible protein band was individually excised from the gel and subjected to polypeptide mass fingerprint analysis, which was carried out by the *BGI Co*., Ltd Company (Shenzhen, China, website: http://www.bgi.com/us/). The putative mycovirus proteins and proteomic data of *S*. *sclerotiorum* were used as the reference database to identify the viral polypeptides.

### Horizontal transmission of virus between *S*. *sclerotiorum* strains belonging to different incompatibility groups

To evaluate efficacy of horizontal transmission of mycoviruses between *S*. *sclerotiorum* strains belonging to different VCGs, mycovirus-infected strains were individually co-cultured with four vegetatively (somatic) incompatible *S*. *sclerotiorum* strains on a PDA plate (15 cm in diameter) for 2 days at 20°C and then 5 days at 10°C. Mycelial agar plugs were picked up from the margin areas of five recipient strains (at sites farthest from the mycovirus-infected strains), and then transferred to a fresh PDA plate. The efficiency of mycovirus horizontal transmission was assayed by RT-PCR amplification and dsRNA extraction. Sixty PDA plates of individual strains were used in horizontal transmission assays.

### Assays of biological properties

The biological properties including hyphal growth rate, colony morphology, and virulence on host plants were evaluated as previously described [48]. Each treatment was replicated at least four times and all biological characterization experiments were conducted at least twice. To assay tolerance to stress of strains Ep-1PNA367 and Ep-1PNA367T1, the two strains were cultured on PDA plates containing different H_2_O_2_ concentrations (0 mM, 7.5 mM, 15 mM, 20 mM) and Sorbitol (1M) under dark conditions, or incubated at different temperatures (5°C, 10°C, 20°C, 30°C, or 35°C) on PDA plates. All plates were then measured for growth rates and the data collected was used for evaluation of tolerance to stress. To define whether infection with SsMYRV4 benefits transmission of other mycoviruses and disease management in living plants, mycelial suspensions of each hypovirulent strain were sprayed on two month old plants of *B*. *napus* and *N*. *benthamiana*, and then the treated plants were inoculated with a virulent strain. Briefly, 2 g wet mycelia of Ep-1PN, Ep-1PNA367T1 and Ep-A367T1 suspended in 40 ml sterile H_2_O were sprayed on plant leaves. This was followed 3 days later, by spraying strain Ep-1PNA367R and 1980R (2 g/40ml). The inoculated plants were placed in an incubator at 20–22°C and 100% relative humidity. Photographs were taken 5 days post-inoculation.

Experimental data were subjected to statistical analysis of variance using the SAS program (ftp://legacy.gsfc.nasa.gov/xmm/software/sas/). Treatment means were compared using the least significant difference test at P = 0.05 level.

### Evans blue staining, NBT staining and hydrogen peroxide measurement

To detect the PCD reaction between two vegetatively incompatible strains, Evans Blue staining was performed as previously described [75]. Briefly, two *S*. *sclerotiorum* strains were dual-cultured on CM-PDA plates for 2 days, when hyphae from the two strains intertwined at the interface region; the latter was stained for 30 min in a 0.5% (w/v) solution of Evans Blue, and then softly washed with ddH_2_O. The result of color reaction was recorded by photography and used for evaluating the PCD reaction.

The NBT staining was used for qualitative detection of ROS level in mycelia of *S*. *sclerotiorum* strains as previously described with some modifications [76]. Briefly, *S*. *sclerotiorum* strains were cultured on CM-PDA plates for 2 days. The colony of each strain was stained with 0.5% NBT in 1 mol/L phosphate buffer solution (pH = 7.4) for 1 hour under dark conditions, and then the reaction was terminated with anhydrous ethanol. H_2_O_2_ production of strains was evaluated based on color reaction and photographed under stereoscope (Olympus SZX16). For quantitative measurement of H_2_O_2_ level in mycelia of the different *S*. *sclerotiorum* strains, the strains were cultured for 3–4 days and then mycelia were harvested. H_2_O_2_ detection was made following the detailed protocol in the manufacturer’s manual (Beyotime Biotechnology, China).

### RT-PCR amplification, qPCR and transcriptional analysis

To assay mycovirus content or gene expression levels in individual strains, mycelia were collected and then used for total RNA extraction and cDNA synthesis. To detect the expression of *het* (*vic*) or G protein subunit genes in SsMYRV4-infected/free strains when contacted with vegetatively incompatible strains, strains Ep-1PNA367 and Ep-1PNA367T1 were each dual-cultured with strain 1980 on CM-PDA plates. The mycelia of Ep-1PNA367 and Ep-1PNA367T1 were collected when the mycelial tips of each strain contacted with that of strain 1980 in 3 hours. Strains Ep-1PNA367 and Ep-1PNA367T1 were cultured and collected alone as controls. To detect the expression of G protein subunit genes in SsMYRV4-free strains 1980 or RL19 when contacted with vegetatively incompatible strain Ep-1PNA367T1, the mycelia of 1980 or RL19 were collected when the mycelial tips of each strain contacted with that of strain Ep-1PNA367T1 in 3 hours. cDNA was used as a template for RT-PCR or qPCR. Total cDNA abundance was normalized using the *Ss-actin* gene as an internal control. The primers used for RT-PCR and qPCR analyses are listed in S5 Table.

For RNA-seq analysis, fresh mycelia of strains Ep-1PNA367 and Ep-1PNA367T1 were grown on a cellophane membrane overlaid onto PDA medium at 20°C, and samples were collected for total RNA extraction at 72 hour. The RNA samples were sequenced and analyzed by the *BGI Co*., Ltd (Shenzhen, China, website: http://www.bgi.com/us/). The raw reads produced by the Illumina HiSeq 2000 were next subjected to quality control. Data analysis was accomplished in two sequential tasks: (1) The RNA_Seq raw sequences were transformed into clean tags and subsequently mapped to the *S*. *sclerotiorum* transcript database from the Broad Institute (http://www.broadinstitute.org/annotation/genome/sclerotinia_sclerotiorum/MultiDownloads.html). (2) count the read fragments mapped to each individual gene and quantify expression by the corresponding RPKM (Reads Per Kilobase per Million mapped reads). The relative expression of HET conserved domain-contained genes in the RNA-Seq data were identified with the threshold of RPKM value.

## Supporting information

S1 Fig Biological characteristics of SsMYRV4-free strain Ep-1PNA367, and SsMYRV4-infected strains SX10 and Ep-1PNA367T1. (A) Growth rate of three strains under laboratory conditions at 20°C. (B) Lesion diameters induced by the three strains on detached rapeseed leaves (20°C, 3 days post-inoculation). (C and D) Sclerotia of strains Ep-1PNA367 and Ep-1PNA367T1. Sclerotia from four PDA plates of each strain were collected and photographed together. The sclerotia yield of strains Ep-1PNA367 and Ep-1PNA367T1 was compared. The symbol “*” indicates significant differences (P = 0.05) among strains of *S*. *sclerotiorum* according to the Student t test.
(TIF)

S2 Fig Transfection of protoplasts of *S*. *sclerotiorum* virulent strain Ep-1PNA367 with purified SsMYRV4 particles. Comparison of colony morphologies (A) and hyphal growth rates (C) of parental strain Ep-1PNA367, strain Ep-1PNA367T1 (derived from a dual culture of strains Ep-1PNA367 and SX10), and the SsMYRV4-transfected isolates Ep-1PNA367PT1. All strains were cultured at 20°C on PDA plates for 48 hour prior to photograph. Virulence assay (B) and lesion length (D) caused by strains Ep-1PNA367, strain Ep-1PNA367T1, and Ep-1PNA367PT1 (20°C, 3 days post- inoculation) on the leaves of rapeseed. (E) dsRNA extraction of SsMYRV4 from individual isolates. Results in each of the histograms shown in panels B to D are expressed as arithmetic means ± standard errors of the means. Asterisks indicate a significant difference (P = 0.05) among strains of *S*. *sclerotiorum* according to the Student t test.
(TIF)

S3 Fig Genome size and organization of SsMYRV4. SsMYRV4 comprises 12 dsRNA segments. ORFs encoded by each dsRNA were boxed, and labeled with viral protein numbers (VP1 to VP12). The largest ORF encodes RNA dependent RNA polymerase (RdRp) protein (VP1). The putative coat proteins (VP2, VP3, VP5, and VP6) were shaded in yellow color. Note that the first seven nucleotides (ACAATTT) and last six nucleotides (GCAGAC) of each dsRNA segments are highly conserved.
(PDF)

S4 Fig Amino acid sequence alignment of core RdRp motifs of SsMYRV4 and selected viruses from family *Reoviridae*. The positions of the conserved motifs are shaded. Numbers in parenthesis refer to amino acids in the intervals between conserved motifs. Seven motifs (I to VII) are detected in the sequence of the conserved RdRp region. Identical residues are indicated by asterisks; conserved and semiconserved amino acid residues are indicated by colons and dots, respectively.
(TIF)

S5 Fig SsMYRV4-mediated horizontal transmission of hypovirulence-associated heterologous viruses and consequent control of Sclerotinia diseases. (A) SsMYRV4-mediated enhancement of SsDRV transmission with resultant control of Sclerotinia diseases on plants of *N*. *benthamiana*. 2 g wet mycelia of Ep-1PN (infected with SsDRV) and Ep-A367T1 (co-infected with SsRV-L, SsDRV and SsMYRV4) suspended in 40 ml sterile H_2_O were sprayed on *N*. *benthamiana* leaves. This was followed 3 days later, by spraying strain Ep-1PNA367R and 1980R (2 g/40ml). The inoculated plants were placed in an incubator at 20–22°C and 100% relative humidity. Photographs were taken at 5 dpi. (B) An isolate of 1980R was derived from a diseased stalk pre-treated with Ep-A367T1 and purified by culturing on 30μg/ml hygromycin PDA medium. (C) Mycovirus content was determined by RT-PCR amplification. Primers for SsMYRV4, SsDRV and SsRV-L were listed in S5 Table.
(TIF)

S6 Fig SsMYRV4-mediated enhancement of tolerance to environmental stress in *S*. *sclerotiorum*. (A) Hyphal growth inhibition of SsMYRV4-infected Ep-1PNA367T1 and SsMYRV4-free strain Ep-1PNA367 were assayed under different temperatures (5°C, 10°C, 30°C, or 35°C). (B) SsMYRV4-infected strain showed stronger tolerance to osmotic stress (1M sorbitol). (C) Colony morphology of strains Ep-1PNA367 and Ep-1PNA367T1 at different conditions.
(TIF)

S1 Table Accession numbers of SsMYRV4 and sequence information compared with RnMYRV3. (DOCX)

S2 Table List of *Reoviridae* members used in phylogenetic analysis. (DOCX)

S3 Table Peptide mass fingerprinting analysis of purified particles preparations. (DOCX)

S4 Table Transmission efficiency of SsDRV and SsRV-L between different VCGs in presence or absence of SsMYRV4. (DOCX)

S5 Table Primers used in this study. (DOCX)
