# Supplementary figures and images for: Virus-mediated suppression of host non-self recognition facilitates horizontal transmission of heterologous viruses
Source: PLoS Pathog. 2017 Mar 23;13(3):e1006234. doi: 10.1371/journal.ppat.1006234 (PMC5363999; doi:10.1371/journal.ppat.1006234)

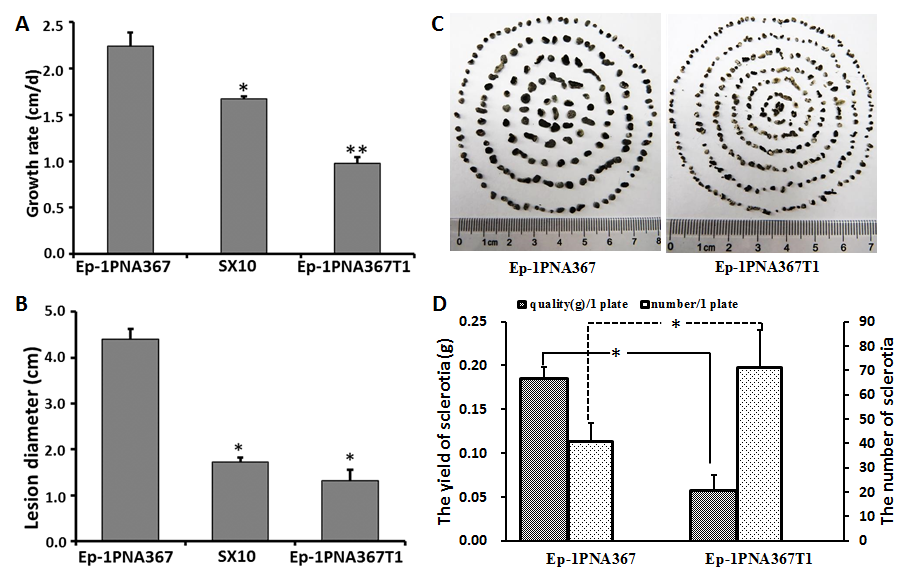

Supplement: S1 Fig — (A) Growth rate of three strains under laboratory conditions at 20°C. (B) Lesion diameters induced by the three strains on detached rapeseed leaves (20°C, 3 days post-inoculation). (C and D) Sclerotia of strains Ep-1PNA367 and Ep-1PNA367T1. Sclerotia from four PDA plates of each strain were collected and photographed together. The sclerotia yield of strains Ep-1PNA367 and Ep-1PNA367T1 was compared. The symbol “*” indicates significant differences (P = 0.05) among strains of S. sclerotiorum according to the Student t test. (TIF) [file ppat.1006234.s001.tif]

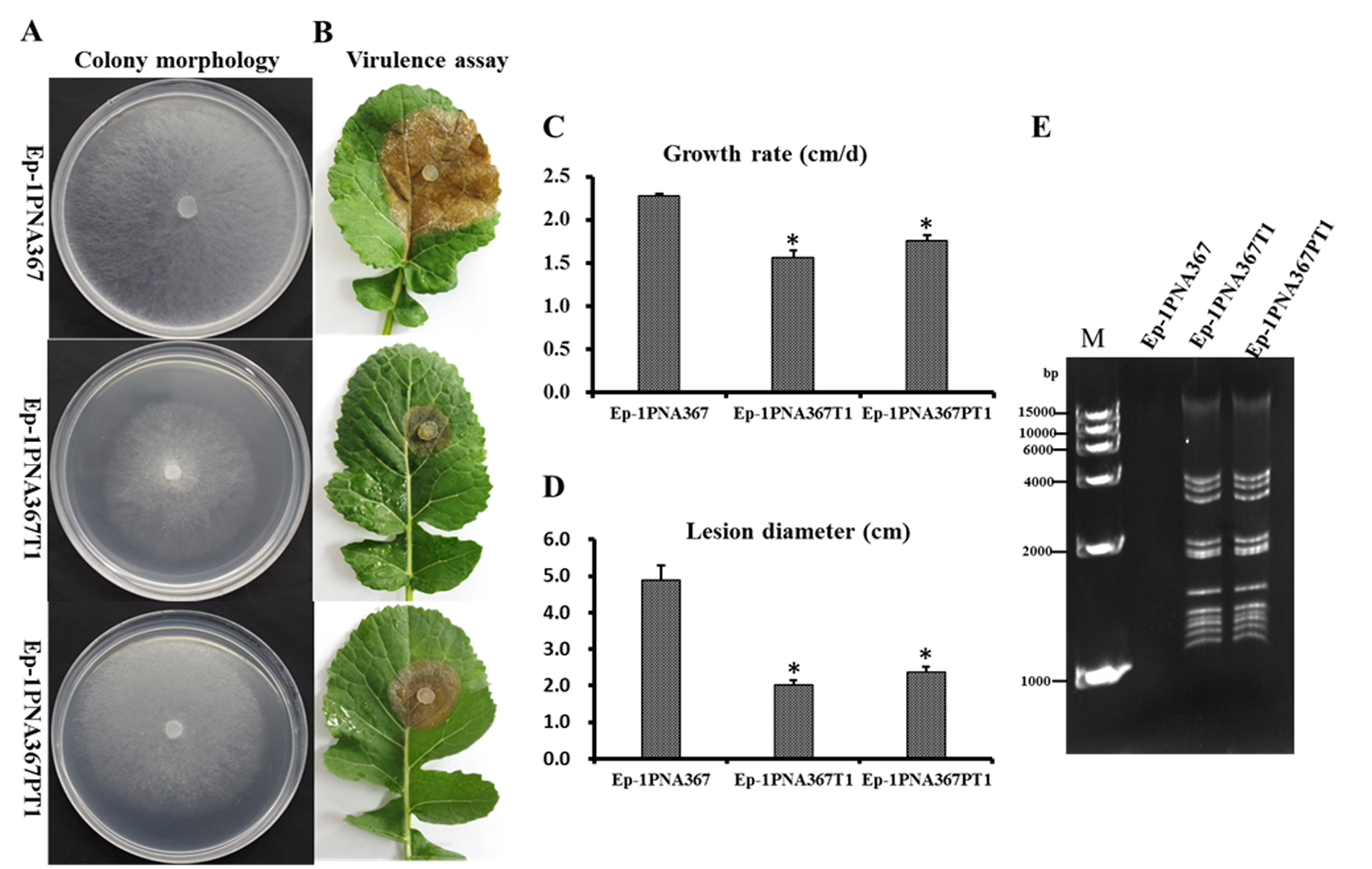

Supplement: S2 Fig — Comparison of colony morphologies (A) and hyphal growth rates (C) of parental strain Ep-1PNA367, strain Ep-1PNA367T1 (derived from a dual culture of strains Ep-1PNA367 and SX10), and the SsMYRV4-transfected isolates Ep-1PNA367PT1. All strains were cultured at 20°C on PDA plates for 48 hour prior to photograph. Virulence assay (B) and lesion length (D) caused by strains Ep-1PNA367, strain Ep-1PNA367T1, and Ep-1PNA367PT1 (20°C, 3 days post- inoculation) on the leaves of rapeseed. (E) dsRNA extraction of SsMYRV4 from individual isolates. Results in each of the histograms shown in panels B to D are expressed as arithmetic means ± standard errors of the means. Asterisks indicate a significant difference (P = 0.05) among strains of S. sclerotiorum according to the Student t test. (TIF) [file ppat.1006234.s002.tif]

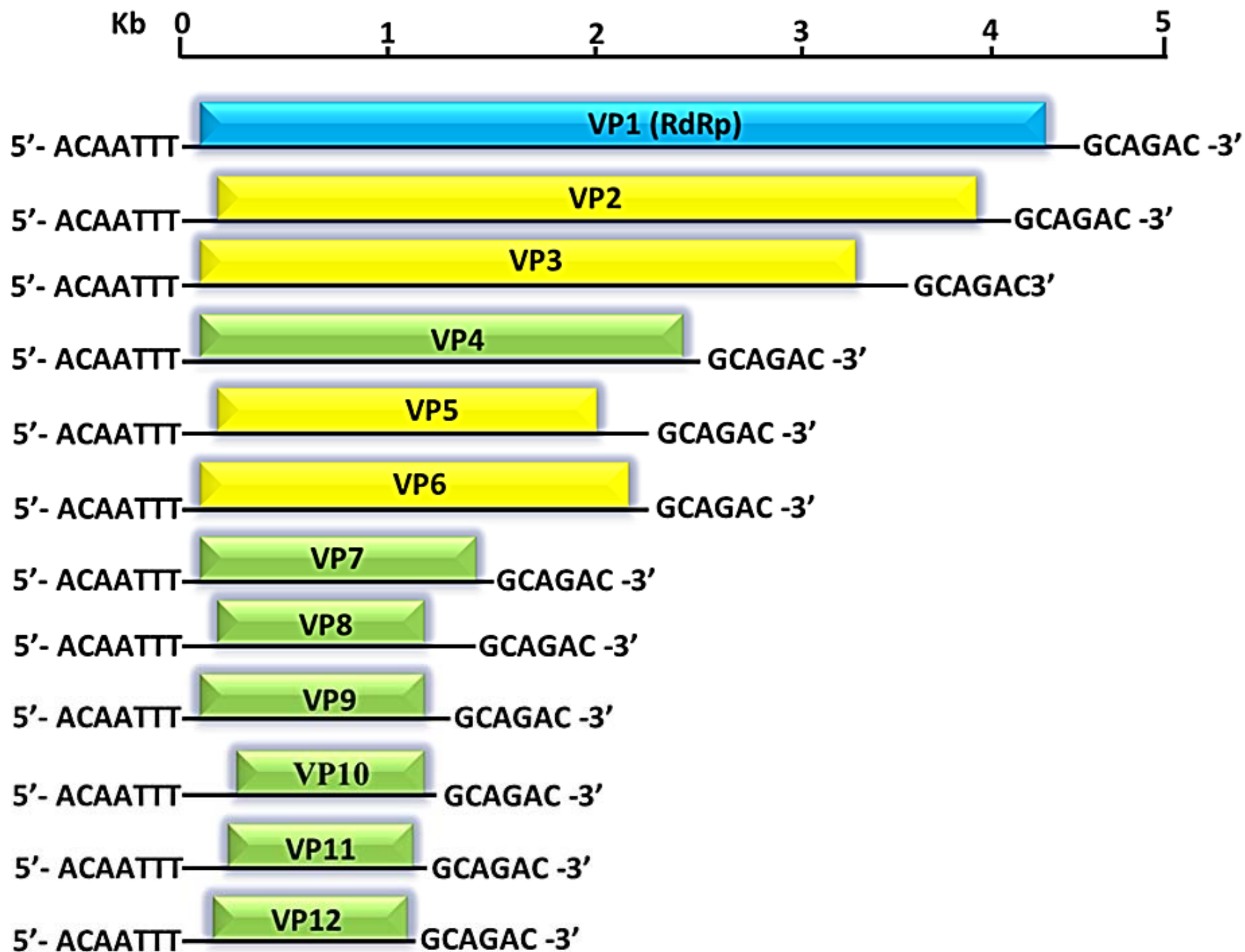

Supplement: S3 Fig — SsMYRV4 comprises 12 dsRNA segments. ORFs encoded by each dsRNA were boxed, and labeled with viral protein numbers (VP1 to VP12). The largest ORF encodes RNA dependent RNA polymerase (RdRp) protein (VP1). The putative coat proteins (VP2, VP3, VP5, and VP6) were shaded in yellow color. Note that the first seven nucleotides (ACAATTT) and last six nucleotides (GCAGAC) of each dsRNA segments are highly conserved. (PDF) [file ppat.1006234.s003.pdf]

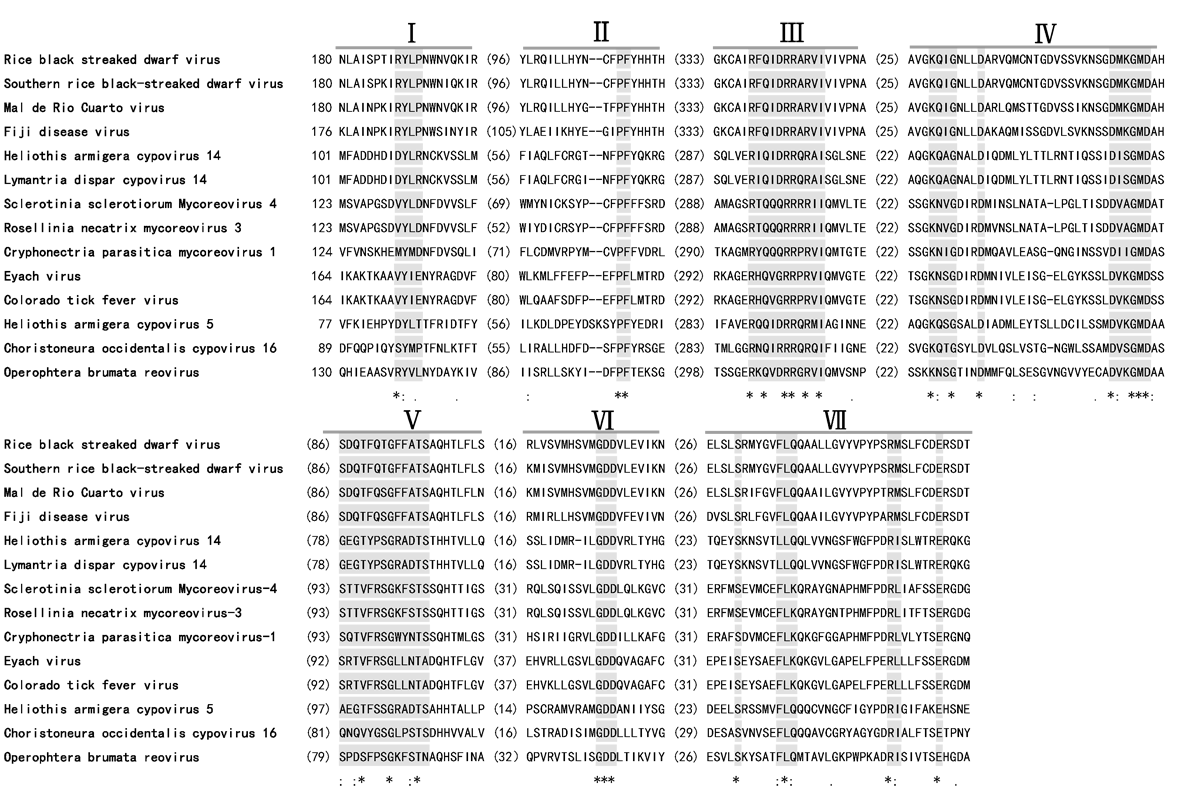

Supplement: S4 Fig — The positions of the conserved motifs are shaded. Numbers in parenthesis refer to amino acids in the intervals between conserved motifs. Seven motifs (I to VII) are detected in the sequence of the conserved RdRp region. Identical residues are indicated by asterisks; conserved and semiconserved amino acid residues are indicated by colons and dots, respectively. (TIF) [file ppat.1006234.s004.tif]

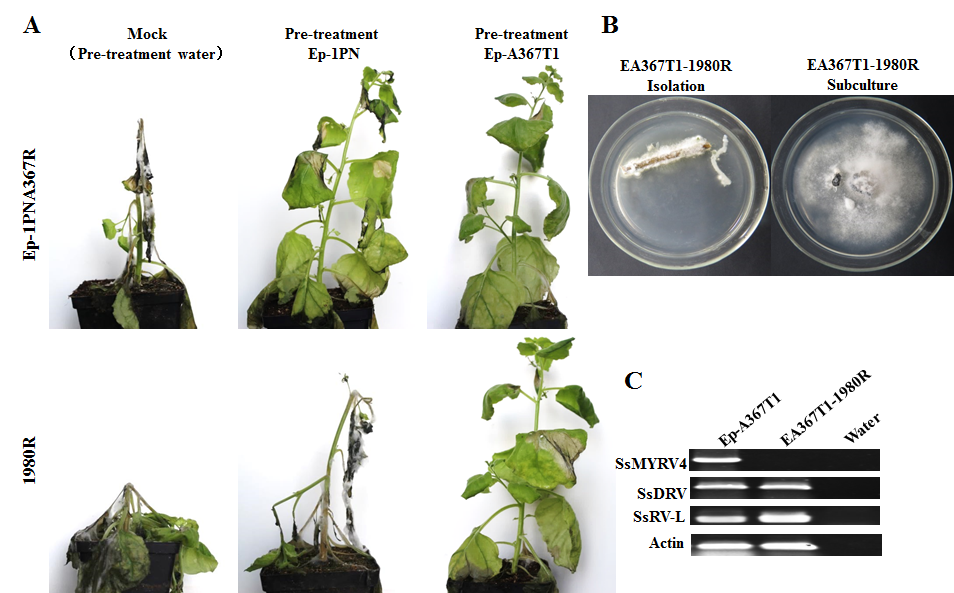

Supplement: S5 Fig — (A) SsMYRV4-mediated enhancement of SsDRV transmission with resultant control of Sclerotinia diseases on plants of N. benthamiana. 2 g wet mycelia of Ep-1PN (infected with SsDRV) and Ep-A367T1 (co-infected with SsRV-L, SsDRV and SsMYRV4) suspended in 40 ml sterile H2O were sprayed on N. benthamiana leaves. This was followed 3 days later, by spraying strain Ep-1PNA367R and 1980R (2 g/40ml). The inoculated plants were placed in an incubator at 20–22°C and 100% relative humidity. Photographs were taken at 5 dpi. (B) An isolate of 1980R was derived from a diseased stalk pre-treated with Ep-A367T1 and purified by culturing on 30μg/ml hygromycin PDA medium. (C) Mycovirus content was determined by RT-PCR amplification. Primers for SsMYRV4, SsDRV and SsRV-L were listed in S5 Table. (TIF) [file ppat.1006234.s005.tif]

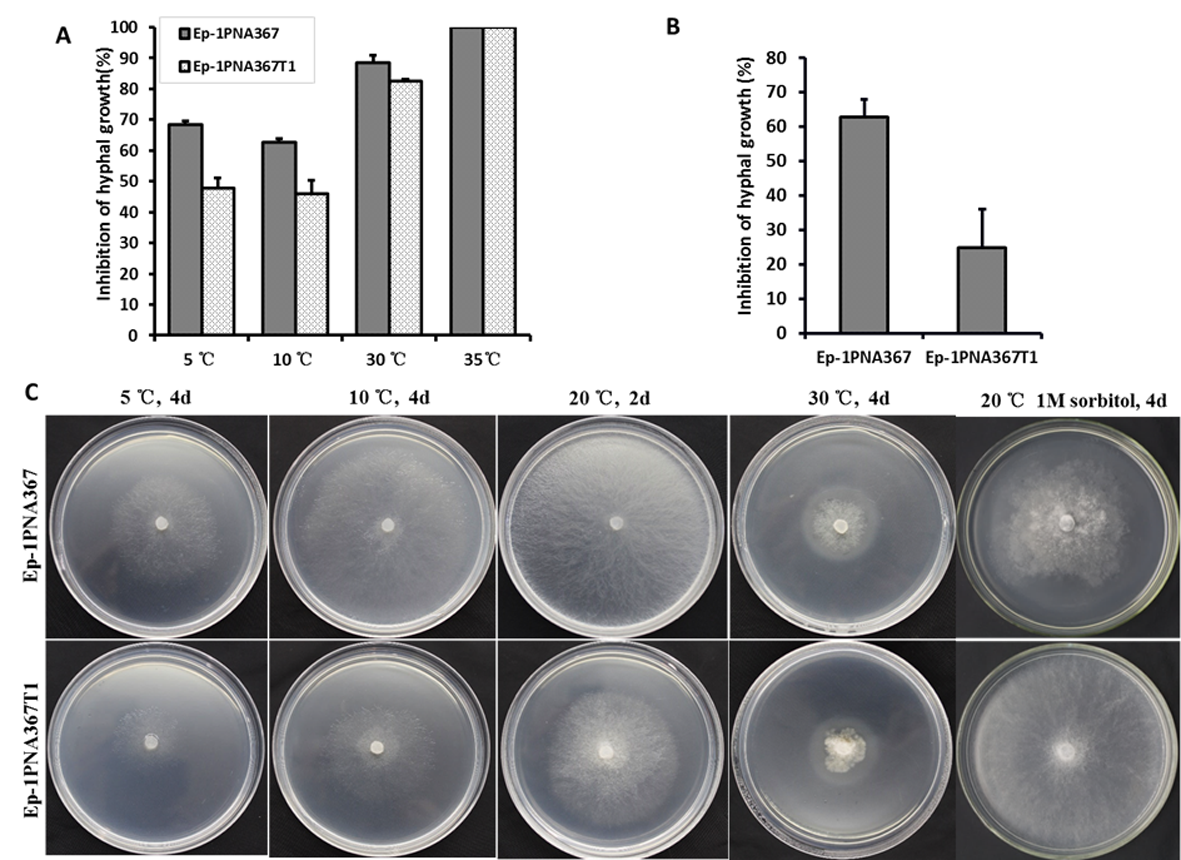

Supplement: S6 Fig — (A) Hyphal growth inhibition of SsMYRV4-infected Ep-1PNA367T1 and SsMYRV4-free strain Ep-1PNA367 were assayed under different temperatures (5°C, 10°C, 30°C, or 35°C). (B) SsMYRV4-infected strain showed stronger tolerance to osmotic stress (1M sorbitol). (C) Colony morphology of strains Ep-1PNA367 and Ep-1PNA367T1 at different conditions. (TIF) [file ppat.1006234.s006.tif]
