# Supplementary material for: Virus-mediated suppression of host non-self recognition facilitates horizontal transmission of heterologous viruses
Source: PLoS Pathog. 2017 Mar 23;13(3):e1006234. doi: 10.1371/journal.ppat.1006234 (PMC5363999; doi:10.1371/journal.ppat.1006234)
Supplement: S1 Table — (DOCX) [file ppat.1006234.s007.docx]

S1 Table. Accession numbers of SsMYRV-4 and sequence information compared with RnMYRV-3.

| **SsMYRV-4** | **Length**  **(bp)** | **Genbank Acc.no.** | **RnMYRV-3** | **Length**  **(bp)** | **Blastn** | | **Blastp** | |
| --- | --- | --- | --- | --- | --- | --- | --- | --- |
|  |  |  |  |  | **coverage** | **identity** | **coverage** | **identity** |
| **S_1_** | **4143** | **KU128375** | **S_1_** | **4143** | **100%** | **77%** | **100%** | **91%** |
| **S_2_** | **3775** | **KU128376** | **S_2_** | **3773** | **100%** | **75%** | **100%** | **87%** |
| **S_3_** | **3307** | **KU128377** | **S_3_** | **3310** | **100%** | **73%** | **100%** | **82%** |
| **S_4_** | **2259** | **KU128378** | **S_4_** | **2259** | **100%** | **78%** | **100%** | **92%** |
| **S_5_** | **2084** | **KU128379** | **S_5_** | **2089** | **100%** | **78%** | **100%** | **83%** |
| **S_6_** | **2029** | **KU128380** | **S_6_** | **2030** | **64%** | **74%** | **89%** | **76%** |
| **S_7_** | **1512** | **KU128381** | **S_7_** | **1509** | **72%** | **76%** | **100%** | **69%** |
| **S_8_** | **1304** | **KU128382** | **S_8_** | **1299** | **100%** | **77%** | **72%** | **72%** |
| **S_9_** | **1223** | **KU128383** | **S_9_** | **1226** | **100%** | **67%** | **100%** | **57%** |
| **S_10_** | **1170** | **KU128384** | **S_10_** | **1171** | **100%** | **80%** | **99%** | **84%** |
| **S_11_** | **1093** | **KU128385** | **S_12_** | **943** | **3%** | **95%** | **96%** | **40%** |
| **S_12_** | **1000** | **KU128386** | **S_11_** | **1003** | **100%** | **76%** | **100%** | **81%** |
