# Supplementary material for: Virus-mediated suppression of host non-self recognition facilitates horizontal transmission of heterologous viruses
Source: PLoS Pathog. 2017 Mar 23;13(3):e1006234. doi: 10.1371/journal.ppat.1006234 (PMC5363999; doi:10.1371/journal.ppat.1006234)
Supplement: S2 Table — (DOCX) [file ppat.1006234.s008.docx]

S2 Table. List of *Reoviridae* members used in phylogenetic analysis.

| **Virus name** | **RdRp size**  **(aa)** | **Identity** | **E-value** | **Genbank Acc.no.** | **Genus** |
| --- | --- | --- | --- | --- | --- |
| Mycoreovieus 4 | 1360 | - | - | [NC_030158.1](http://www.ncbi.nlm.nih.gov/nuccore/1028356610) | Mycoreovirus |
| Mycoreovirus3 | 1360 | 91% | 0 | [YP_392478.1](http://www.ncbi.nlm.nih.gov/protein/80540094?report=genbank&log$=prottop&blast_rank=1&RID=77UY5REB01R) |  |
| Mycoreovieus 1 | 1354 | 38% | 0 | [YP_001936004.1](http://www.ncbi.nlm.nih.gov/protein/189022225?report=genbank&log$=prottop&blast_rank=2&RID=77UY5REB01R) |  |
| Eyach virus | 1435 | 29% | 1e-150 | [AAM18358.1](http://www.ncbi.nlm.nih.gov/protein/20270664?report=genbank&log$=prottop&blast_rank=6&RID=77UY5REB01R) | Coltivirus |
| Colorado tick fever virus | 1435 | 29% | 1e-150 | [AAM18357.1](http://www.ncbi.nlm.nih.gov/protein/20270662?report=genbank&log$=prottop&blast_rank=8&RID=77UY5REB01R) |  |
| Southern rice streaked dwarf virus | 1464 | 22% | 5e-9 | [CCW59432.1](http://www.ncbi.nlm.nih.gov/protein/647393709?report=genbank&log$=prottop&blast_rank=16&RID=4JXX3YSU01R) | Fijivirus |
| Rice black streaked dwarf virus | 1464 | 22% | 9e-10 | [AFX68406.1](http://www.ncbi.nlm.nih.gov/protein/418972440?report=genbank&log$=prottop&blast_rank=15&RID=77UY5REB01R) |  |
| Mal de Rio Cuarto virus | 1464 | 23% | 3e-05 | [AAY51840.1](http://www.ncbi.nlm.nih.gov/protein/66731260?report=genbank&log$=prottop&blast_rank=30&RID=77UY5REB01R) |  |
| Fiji diseases virus | 1470 | 22% | 6e-04 | [YP_249762.1](http://www.ncbi.nlm.nih.gov/protein/68532385?report=genbank&log$=prottop&blast_rank=39&RID=77UY5REB01R) |  |
| Operophtera brumata reovirus | 1358 | 28% | 3e-11 | [YP_392501.1](http://www.ncbi.nlm.nih.gov/protein/78768327?report=genbank&log$=prottop&blast_rank=13&RID=77UY5REB01R) | unclassified *Reoviridae* |
| Choristoneura occidentalis cypovirus 16 | 1225 | 21% | 0.008 | [ACA53380.1](http://www.ncbi.nlm.nih.gov/protein/169313610?report=genbank&log$=prottop&blast_rank=40&RID=77UY5REB01R) | Cypovirus |
| Cypovirus 5 | 1208 | 23% | 5e-7 | [YP_001883321.1](http://www.ncbi.nlm.nih.gov/protein/187765472?report=genbank&log$=prottop&blast_rank=1&RID=77XNW18901R) |  |
| Heliothis armigera cypovirus 14 | 1228 | 22% | 2e-04 | [ABB51571.1](http://www.ncbi.nlm.nih.gov/protein/78924361?report=genbank&log$=prottop&blast_rank=36&RID=77UY5REB01R) |  |
| Cypovirus 14 | 1228 | 22% | 5e-4 | [NP_149135.1](http://www.ncbi.nlm.nih.gov/protein/14993611?report=genbank&log$=prottop&blast_rank=38&RID=77UY5REB01R) |  |
