# Supplementary material for: Virus-mediated suppression of host non-self recognition facilitates horizontal transmission of heterologous viruses
Source: PLoS Pathog. 2017 Mar 23;13(3):e1006234. doi: 10.1371/journal.ppat.1006234 (PMC5363999; doi:10.1371/journal.ppat.1006234)
Supplement: S3 Table — (DOCX) [file ppat.1006234.s009.docx]

| Sample name | Amino acid sequence  S3 Table. Peptide mass fingerprinting analysis of purified particles preparations. | Mass | | ±Δ | Putative ORF  / Coverage | Position | Frequency |
| --- | --- | --- | --- | --- | --- | --- | --- |
|  |  | Calculated Observed | |  |  |  |  |
| **P138** | ALPWGYR | 861.53 | 861.45 | 0.0791 | **P2: 49.02%** | 552-558 | 1 |
|  | DPTRYSR | 893.52 | 893.44 | 0.0846 |  | 621-627 | 1 |
|  | ATILLDFIR | 1060.71 | 1060.63 | 0.0796 |  | 531-53 | 1 |
|  | VYTQSPVR | 948.59 | 948.5 | 0.0845 |  | 1208-1215 | 1 |
|  | HLGFLGADR | 984.6 | 984.51 | 0.0883 |  | 877-885 | 1 |
|  | QDAFSLSAVK | 1048.57 | 1048.51 | 0.059 |  | 81-90 | 1 |
|  | DLLGVLSVLR | 1083.75 | 1083.67 | 0.0827 |  | 349-358 | 1 |
|  | NYYFGTDPR | 1131.59 | 1131.5 | 0.0915 |  | 809-817 | 1 |
|  | LFDLIGVSFR | 1165.74 | 1165.65 | 0.0892 |  | 217-226 | 1 |
|  | FTLLEPIPPR | 1181.77 | 1181.68 | 0.0898 |  | 582-591 | 1 |
|  | ANFEFLTEMR | 1256.65 | 1256.59 | 0.0654 |  | 329-338 | 1 |
|  | GFQLSPDAYR | 1152.65 | 1152.56 | 0.0911 |  | 339-348 | 1 |
|  | KPILSADVTGPR | 1252.81 | 1252.71 | 0.0922 |  | 1137-1148 | 1 |
|  | VIVEGLATSVLR | 1255.81 | 1255.75 | 0.0553 |  | 416-427 | 1 |
|  | EPTEAWESFGR | 1307.67 | 1307.58 | 0.092 |  | 26-26 | 1 |
|  | KYDVVLDTFGR | 1311.79 | 1311.68 | 0.1047 |  | 372-382 | 1 |
|  | DHYFVLSLPNR | 1359.77 | 1359.69 | 0.0802 |  | 1068-1078 | 1 |
|  | ILVDGWNTADAR | 1329.76 | 1329.67 | 0.0877 |  | 1149-1160 | 1 |
|  | VNPYDLSDTVIR | 1390.8 | 1390.71 | 0.0913 |  | 171-182 | 1 |
|  | IAFLMNPLAYDR | 1422.82 | 1422.73 | 0.0892 |  | 514-525 | 2 |
|  | ELTPAFALVTIFNR | 1591.95 | 1591.86 | 0.0913 |  | 653-666 | 1 |
|  | HLGFLGADRGLGK | 1339.67 | 1339.74 | -0.064 |  | 877-889 | 1 |
|  | NALDPAGWNLMTR | 1457.8 | 1457.71 | 0.0892 |  | 592-604 | 1 |
|  | LACDFTLLYSAQFR | 1703.93 | 1703.83 | 0.0909 |  | 398-411 | 1 |
|  | HPGIVVFETVEDLLR | 1723.02 | 1722.93 | 0.0923 |  | 1017-1031 | 1 |
|  | VTYNEIVIDMILQGLK | 1847.97 | 1848.01 | -0.0343 |  | 1052-1067 | 2 |
|  | VLAFLNAPGLLFDVQR | 1772.09 | 1772 | 0.0921 |  | 605-620 | 1 |
|  | IHANSSLPLSTPAFSIR | 1810.09 | 1809.97 | 0.1119 |  | 64-80 | 1 |
|  | MADSTPLDPLQSSDLK | 1716.96 | 1716.82 | 0.1346 |  | 1-16 | 1 |
|  | VMTPLDENAQEYPVAR | 1831.98 | 1831.88 | 0.0997 |  | 637-652 | 1 |
|  | MADSTPLDPLQSSDLKK | 1845.96 | 1845.9 | 0.0601 |  | 1-17 | 2 |
|  | SILQVGHVCGETIDLVGR | 1952.11 | 1952.01 | 0.0972 |  | 825-842 | 1 |
|  | RQDAYTAFPFGFVAPYR | 2005.08 | 2004.98 | 0.0964 |  | 1191-1207 | 1 |
|  | CLALELMPDWYDISTPR | 2079.08 | 2078.98 | 0.1043 |  | 477-493 | 1 |
|  | HFVQYAAMMPHHVTSLAR | 2095.1 | 2095.02 | 0.0767 |  | 1080-1097 | 3 |
|  | DDILTANSISAVFNQLAATR | 2119.19 | 2119.09 | 0.1016 |  | 40-59 | 2 |
|  | YPHYPEVIVMNHHLDWVR | 2305.26 | 2305.11 | 0.1512 |  | 270-287 | 1 |
|  | YFCGALNPSATGVDWDNVR | 2141.08 | 2140.96 | 0.1161 |  | 790-808 | 1 |
|  | VDGQAPDFASYFPDVLEGDDGR | 2369.18 | 2369.04 | 0.1323 |  | 1161-1182 | 1 |
|  | LLVDAVFSPELGVLPVLDDVNVSYR | 2728.59 | 2728.47 | 0.1214 |  | 938-962 | 1 |
|  | ADGIEEIDMGQYSGGLVTQNDMNGR | 2669.29 | 2669.17 | 0.1208 |  | 183-207 | 1 |
|  | DSHNVINTTYGQVGFELIGLSIDTSIER | 3077.68 | 3077.53 | 0.1501 |  | 446-473 | 1 |
|  | VVEHVAIATPEHVPHFYALRPDLDMPK | 3080.8 | 3080.59 | 0.214 |  | 91-117 | 2 |
| **P121** | VVECLR | 774.42 | 774.41 | 0.0143 | **P3: 40.92%** | 298-303 | 1 |
|  | SLDFAIR | 820.46 | 820.44 | 0.0153 |  | 237-243 | 1 |
|  | YNVDGLR | 835.44 | 835.42 | 0.0189 |  | 155-161 | 1 |
|  | QVFLSLR | 861.53 | 861.51 | 0.0202 |  | 275-281 | 1 |
|  | LGDELAIR | 885.51 | 885.49 | 0.0135 |  | 820-827 | 1 |
|  | NQWTSFK | 910.46 | 910.42 | 0.0412 |  | 363-369 | 1 |
|  | VIETLFNR | 990.56 | 990.55 | 0.0105 |  | 677-684 | 1 |
|  | AQTASGSVR | 875.46 | 875.45 | 0.0164 |  | 584-592 | 1 |
|  | ENYVELFHR | 1205.59 | 1205.58 | 0.0066 |  | 354-362 | 1 |
|  | ADWYVNVR | 1021.51 | 1021.5 | 0.0152 |  | 872-879 | 1 |
|  | DPLPVSVYR | 1044.56 | 1044.56 | -0.0009 |  | 414-422 | 2 |
|  | MSGPWVDKR | 1074.54 | 1074.53 | 0.0137 |  | 891-899 | 1 |
|  | WLDHNGHMR | 1164.55 | 1164.52 | 0.0262 |  | 794-802 | 3 |
|  | IREDPLASENR | 1298.65 | 1298.66 | -0.0056 |  | 90-100 | 1 |
|  | LTCVNTPFVVQR | 1432.74 | 1432.75 | -0.0127 |  | 593-604 | 1 |
|  | YVPYNVHFLDAR | 1492.74 | 1492.75 | -0.0063 |  | 222-233 | 1 |
|  | NAPDMPNEVRPR | 1394.67 | 1394.67 | -0.0019 |  | 839-850 | 2 |
|  | YVIVDPGNVVGEAR | 1486.76 | 1486.78 | -0.0144 |  | 900-913 | 1 |
|  | GPAAEYMSSVLMSR | 1497.7 | 1497.7 | 0.0046 |  | 501-514 | 1 |
|  | NANFGFSHVPSYEAR | 1694.76 | 1694.78 | -0.0215 |  | 1055-1069 | 1 |
|  | QLFSDLATTINVVEQR | 1815.91 | 1815.94 | -0.0282 |  | 118-133 | 2 |
|  | ILAVALEYGNAVLDGYR | 1835.95 | 1835.98 | -0.0289 |  | 101-117 | 1 |
|  | TGEPLEFSFVNPGITVR | 1861.93 | 1861.96 | -0.027 |  | 921-937 | 1 |
|  | ESQLAAITAIEHELVGPK | 1904.98 | 1905.02 | -0.0393 |  | 971-988 | 1 |
|  | LENDIESVVTYVDSICQR | 2138.97 | 2139.02 | -0.0472 |  | 332-349 | 1 |
|  | VAFPQVTFDWIGGNGDDIR | 2105.98 | 2106.02 | -0.0399 |  | 516-534 | 1 |
|  | QNFGLLHYFSFSSISDLER | 2242.05 | 2242.07 | -0.0219 |  | 255-273 | 2 |
|  | YYEMISTGASTIPVGEILHIIPR | 2559.31 | 2559.34 | -0.0354 |  | 370-392 | 2 |
|  | APVMSVPLGESTHANAQLVGFK | 2252.11 | 2252.16 | -0.0505 |  | 479-500 | 1 |
|  | VVVHNCGEIQQGHTLADLTDWR | 2547.19 | 2547.23 | -0.0375 |  | 989-1010 | 1 |
|  | SSAFDVPFVNAAIFSILTTAHLTSQGR | 2849.46 | 2849.47 | -0.0073 |  | 557-583 | 1 |
|  | LSTNYEMGVFTEPLLDNGLGVSVACR | 2841.34 | 2841.37 | -0.0243 |  | 685-710 | 1 |
|  | ALPWGYR | 861.53 | 861.45 | 0.0777 | **P2: 16.72%** | 552-558 | 1 |
|  | ATILLDFIR | 1060.63 | 1060.63 | 0.0017 |  | 531-539 | 1 |
|  | FGLSWFLGK | 1053.5 | 1053.56 | -0.0601 |  | 208-216 | 1 |
|  | LFDLIGVSFR | 1165.66 | 1165.65 | 0.0113 |  | 217-226 | 1 |
|  | FTLLEPIPPR | 1181.68 | 1181.68 | -0.0015 |  | 582-591 | 1 |
|  | ILVDGWNTADAR | 1329.79 | 1329.67 | 0.125 |  | 1149-1160 | 1 |
|  | AEEALIRQLMSR | 1432.74 | 1432.73 | 0.0027 |  | 1098-1109 | 1 |
|  | IAFLMNPLAYDRNR | 1694.76 | 1694.84 | -0.0863 |  | 514-527 | 1 |
|  | HPGIVVFETVEDLLR | 1722.91 | 1722.93 | -0.0203 |  | 1017-1031 | 1 |
|  | IRQIVDVIMNSSVLTAK | 1887.93 | 1888.03 | -0.1063 |  | 853-869 | 1 |
|  | VLAFLNAPGLLFDVQR | 1771.98 | 1772 | -0.0193 |  | 605-620 | 1 |
|  | VMTPLDENAQEYPVAR | 1832.93 | 1832.86 | 0.0703 |  | 637-652 | 1 |
|  | MADSTPLDPLQSSDLKK | 1861.93 | 1861.9 | 0.0325 |  | 1-17 | 1 |
|  | TTVIGTPATLNANMTKTR | 1904.98 | 1905 | -0.0175 |  | 428-445 | 1 |
|  | VLAFLNAPGLLFDVQRDPTR | 2242.05 | 2242.21 | -0.1634 |  | 605-624 | 1 |
|  | RHFVQYAAMMPHHVTSLAR | 2252.11 | 2252.11 | 0.0023 |  | 1079-1097 | 1 |
| **P71** | ITDVER | 731.38 | 731.38 | -0.002 | **P5: 39.63%** | 53-58 | 1 |
|  | HLFFIR | 831.48 | 831.48 | 0.0064 |  | 293-298 | 1 |
|  | INSGEIR | 787.42 | 787.42 | 0.0057 |  | 426-432 | 1 |
|  | NSYAWR | 795.38 | 795.37 | 0.0109 |  | 71-76 | 1 |
|  | DVLEVIR | 842.5 | 842.49 | 0.0097 |  | 61-67 | 3 |
|  | LGQYCGR | 852.4 | 852.39 | 0.0045 |  | 472-478 | 1 |
|  | FDHSPSGR | 901.42 | 901.4 | 0.015 |  | 285-292 | 1 |
|  | VACSNQLR | 946.48 | 946.47 | 0.011 |  | 368-75 | 1 |
|  | VDDISTLMR | 1048.53 | 1048.52 | 0.01 |  | 171-179 | 1 |
|  | SLHRLMPVVK | 1178.59 | 1178.7 | -0.1058 |  | 96-105 | 1 |
|  | CMEQLCNAIDR | 1408.61 | 1408.59 | 0.0211 |  | 566-576 | 1 |
|  | CPEGVFVNQPR | 1301.62 | 1301.62 | 0.0038 |  | 623-633 | 1 |
|  | MGYLANYYHYTR | 1550.7 | 1550.7 | 0.007 |  | 524-535 | 2 |
|  | ASTSTIMAGSFGR | 1284.62 | 1284.61 | 0.003 |  | 536-548 | 1 |
|  | SLLDQYASSIDEFAR | 1713.83 | 1713.82 | 0.0059 |  | 81-95 | 1 |
|  | DFSGFFCPACGCVYR | 1841.74 | 1841.73 | 0.0075 |  | 549-563 | 1 |
|  | VTYDTQAITQQINTALTR | 2036.07 | 2036.05 | 0.0112 |  | 150-167 | 1 |
|  | WSFESFANTSPDSPSPIR | 2023.94 | 2023.93 | 0.0079 |  | 433-450 | 1 |
|  | LDHVNQQLAQIHASSEDTR | 2161.07 | 2161.05 | 0.0183 |  | 130-148 | 1 |
|  | LDHVNQQLAQIHASSEDTRR | 2317.17 | 2317.15 | 0.0154 |  | 130-149 | 1 |
|  | NNMELLLQGLGVPDVLDGLMR | 2296.19 | 2296.19 | -0.0041 |  | 451-471 | 3 |
|  | GTSVECFSDAIPVINITTGDGTERPR | 2791.36 | 2791.34 | 0.013 |  | 27-52 | 1 |
| **P75** | YADFER | 799.35 | 799.35 | -0.0013 | **P6: 40.03%** | 550-555 | 1 |
|  | TYMVGKK | 841.49 | 841.44 | 0.0521 |  | 442-448 | 1 |
|  | NFHALLR | 869.48 | 869.49 | -0.0089 |  | 163-169 | 1 |
|  | YLEQTSIR | 1008.5 | 1008.52 | -0.0199 |  | 287-294 | 1 |
|  | CVEVYLER | 1066.52 | 1066.51 | 0.0105 |  | 47-54 | 1 |
|  | SFISHHGVR | 1038.51 | 1038.54 | -0.022 |  | 559-567 | 1 |
|  | YVDVVSNYR | 1113.53 | 1113.55 | -0.0159 |  | 63-71 | 1 |
|  | ATGAQFNVAR | 1033.52 | 1033.53 | -0.0142 |  | 266-275 | 1 |
|  | NWTEGFSLSK | 1167.55 | 1167.56 | -0.0015 |  | 427436 | 1 |
|  | YVDVVSNYRK | 1241.62 | 1241.64 | -0.0253 |  | 63-72 | 1 |
|  | AVPHIFVVNSMEK | 1470.68 | 1470.75 | -0.0767 |  | 78-90 | 2 |
|  | AWNSAMPTQQQR | 1416.64 | 1416.66 | -0.0214 |  | 331-342 | 2 |
|  | LGVPVVDSDDYGR | 1390.65 | 1390.67 | -0.0212 |  | 462-474 | 1 |
|  | VFPPVYLVDLSVSIR | 1702.93 | 1702.97 | -0.0332 |  | 316-330 | 1 |
|  | VLLIAENTGVSLEDAVR | 1797.95 | 1797.98 | -0.0332 |  | 475-491 | 1 |
|  | DCAPTLFETIMDDIVTR | 1995.91 | 1995.93 | -0.0212 |  | 502-518 | 1 |
|  | GQHDNSISELMLAVYYDR | 2109.95 | 2109.98 | -0.0278 |  | 616-633 | 2 |
|  | IHVNIFDLVPTGVVCTVLR | 2151.15 | 2151.19 | -0.0421 |  | 634-652 | 1 |
|  | LQANVDVVYFDDVLTTDGR | 2139.01 | 2139.05 | -0.037 |  | 240-258 | 1 |
|  | SFSHATYTTLPLPTDIYQMLR | 2454.18 | 2454.23 | -0.0406 |  | 26-46 | 2 |
|  | SPHDYVSSDVTVVSNPTSQDIIR | 2515.17 | 2515.22 | -0.0443 |  | 102-124 | 1 |
|  | SPHDYVSSDVTVVSNPTSQDIIRSIR | 2872.31 | 2872.42 | -0.1114 |  | 102-127 | 1 |
| **P45** | ATILLDFIR | 1060.61 | 1060.63 | -0.0216 | **P2: 6.36%** | 531-539 | 1 |
|  | FTLLEPIPPR | 1181.67 | 1181.68 | -0.0124 |  | 582-591 | 1 |
|  | LFDLIGVSFR | 1165.63 | 1165.65 | -0.021 |  | 217-226 | 1 |
|  | DLLGVLSVLR | 1083.64 | 1083.67 | -0.0222 |  | 349-358 | 1 |
|  | EPTEAWESFGR | 1307.56 | 1307.58 | -0.0147 |  | 26-36 | 1 |
|  | NALDPAGWNLMTR | 1474.73 | 1474.69 | 0.0376 |  | 592-604 | 1 |
|  | HPGIVVFETVEDLLR | 1722.9 | 1722.93 | -0.0343 |  | 1017-1031 | 1 |
