# Supplementary material for: Virus-mediated suppression of host non-self recognition facilitates horizontal transmission of heterologous viruses
Source: PLoS Pathog. 2017 Mar 23;13(3):e1006234. doi: 10.1371/journal.ppat.1006234 (PMC5363999; doi:10.1371/journal.ppat.1006234)
Supplement: S4 Table — (DOCX) [file ppat.1006234.s010.docx]

S4 Table Transmission efficiency of SsDRV and SsRV-L between different VCGs in presence or absence of SsMYRV-4.

| **Recipient strains**  **Donor strains** | **Ep-1PNA367** | **1980** | **DT47-39** | **RL19** | **RL26** |
| --- | --- | --- | --- | --- | --- |
| **Ep-1PN**  **(SsDRV and SsRV-L)** | **100%**  **(60/60)** | **0**  **(0/60)** | **0**  **(0/60)** | **0**  **(0/60)** | **5%**  **(3/60)** |
| **Ep-A367T1**  **(SsDRV, SsRV-L and SsMYRV-4)** | **100%**  **(60/60)** | **40%**  **(24/60)** | **30%**  **(18/60)** | **10%**  **(6/60)** | **10%**  **(6/60)** |
