# Supplementary material for: Virus-mediated suppression of host non-self recognition facilitates horizontal transmission of heterologous viruses
Source: PLoS Pathog. 2017 Mar 23;13(3):e1006234. doi: 10.1371/journal.ppat.1006234 (PMC5363999; doi:10.1371/journal.ppat.1006234)
Supplement: S5 Table — (DOCX) [file ppat.1006234.s011.docx]

S5 Table. Primer pairs used in this study.

| **Primer name** | **Primer sequence (5′–3′)** | **Application** | **Product size (bp)** |
| --- | --- | --- | --- |
| Actin-F | CTGGAAGATTGACTGGCGGTTTG | Used as an internal control | 419 |
| Actin-R | AGCACCAGAGGAGCACCCAGTTT |  |  |
| SsMYRV-4-F | CCAGTCGCCAAACTTCTTTATGATC | Detection for SsMYRV-4 | 267 |
| SsMYRV-4-R | GACATTTCCCTTCGCATCAACTCA |  |  |
| SsRV-L-F | CCAGCCTTTCCGACACCGACT | Detection for SsRV-L | 557 |
| SsRV-L-R | GTCAGATTGGCGATAGTGGCGTAA |  |  |
| SsDRV-F | AGCACACACTTCCAGATTCAACTCG | Detection for SsDRV | 508 |
| SsDRV-R | GAGACTTTGACTCATCCAACCATCG |  |  |
| SsMV1-F | GAAGTAGTATTCAAGGACCACCGAG | Detection for SsMV1 | 870 |
| SsMV1-R | CCTGGGATAAAAGTTTTGATTGAGAT |  |  |
| SsActin-F | CTTCCATTGTCGGTCGTCCC | As an endogenous reference for qRT-PCR | 135 |
| SsActin-R | TGACGACACCGTGCATTGGG |  |  |
| Ssnox1F | CTGCTCATGCCAAGATTAGA | Detection of the expression level of  *Ssnox1* | 189 |
| Ssnox1R | CCAACGCCATCCTTCATAT |  |  |
| Ssnox2F | CACTTATGGCTATGGCTATTACT | Detection of the expression level of  *Ssnox2* | 105 |
| Ssnox2R | TGCGGTATCTGGTTGAATC |  |  |
| SS1G_00446F | AGGCGTCCCCGTTGATGAG | Detection of the expression level of  SS1G_00446 | 127 |
| SS1G_00446R | CACGGATTCTGAACCAAGGACA |  |  |
| SS1G_05782F | GGTGGAAGTCTTTTGGCAGGAG | Detection of the expression level of  SS1G_05782 | 118 |
| SS1G_05782R | CCGAGTGACCGACTTCAGTGTAAC |  |  |
| SS1G_05532F | TTGACCGAATCTCTGAACT | detection of the expression level of  SS1G_05532 | 84 |
| SS1G_05532R | GAACGACCTCTACATCTGA |  |  |
| SS1G_04128F | CGTCTACATTCCGATGAG | Detection of the expression level of  SS1G_04128 | 142 |
| SS1G_04128R | CAGCCTATCCTTACAAGTT |  |  |
| SS1G_05625F | GAAGTGAGCAAGGATGTA | Detection of the expression level of  SS1G_05625 | 170 |
| SS1G_05625R | CCGTTGTATCTGGTCTATT |  |  |
| SS1G_02602F | CAGATTGGAGGTTGAGTATA | Detection of the expression level of SS1G_02602 | 103 |
| SS1G_02602R | TTCGTCTAAGTCTTCTAGTG |  |  |
| SS1G_02742F | ACTCGTGTTATGGAACTG | Detection of the expression level of SS1G_02742 | 97 |
| SS1G_02742R | CCTGGTTGTATGACTACTC |  |  |
| SS1G_02744F | CTTATTACGAGGAGCATCA | Detection of the expression level of SS1G_02744 | 174 |
| SS1G_02744R | TGAACACAGTCATCTTGG |  |  |
| SS1G_02890F | GACGATGGATTACTCAAGA | Detection of the expression level of SS1G_02890 | 108 |
| SS1G_02890R | ATCTGAAGCCTCCTAAGT |  |  |
| SS1G_03811F | CAACAAGTGGAATGATGAG | Detection of the expression level of SS1G_03811 | 191 |
| SS1G_03811R | AATGAGACCTAGTAGAATGC |  |  |
| SS1G_03889F | AGCATGTCGTGTAGTTAC | Detection of the expression level of SS1G_03889 | 134 |
| SS1G_03889R | GAATGAGGAGAATGTGTAGA |  |  |
| SS1G_05211F | ACCGAGGAGTTACGATAT | Detection of the expression level of SS1G_05211 | 141 |
| SS1G_05211R | AAGAACAAGTAGATGAGGATT |  |  |
| SS1G_05486F | TTGACCGAATCTCTGAACT | Detection of the expression level of SS1G_05486 | 84 |
| SS1G_05486R | GAACGACCTCTACATCTGA |  |  |
| SS1G_05862F | CCTACTATCACGGTCATAC | Detection of the expression level of SS1G_05862 | 92 |
| SS1G_05862R | GCGAGAAGTAATCTTGTAAC |  |  |
| SS1G_08054F | AAGGATGTATCGTCTTCAC | Detection of the expression level of SS1G_08054 | 162 |
| SS1G_08054R | GCTGTTGTATCTGGTCTAT |  |  |
| SS1G_08974F | TTGACCGAATCTCTGAACT | Detection of the expression level of SS1G_08974 | 84 |
| SS1G_08974R | GAACGACCTCTACATCTGA |  |  |
| SS1G_09167F | CCTGATAACTCTGCTATGAT | Detection of the expression level of SS1G_09167 | 143 |
| SS1G_09167R | CGTCGTATAATCGTATGGA |  |  |
| SS1G_11165F | CAGCAACATATTCCACATC | Detection of the expression level of SS1G_11165 | 170 |
| SS1G_11165R | ATTCATTACACCGACTCTC |  |  |
| SS1G_11315F | AGACAGCATTGAGGAGAG | Detection of the expression level of SS1G_11315 | 82 |
| SS1G_11315R | CGAGCCAGATAATCACTTG |  |  |
| SS1G_13820F | TTACCACAAGGAACCAGAA | Detection of the expression level of SS1G_13820 | 88 |
| SS1G_13820R | GAAGACCGAGAAGTGCTA |  |  |
| SS1G_12343F | CTTCCATCATTCTGTTCTTG | Detection of the expression level of SS1G_12343 | 99 |
| SS1G_12343R | CATCACCACCTTCGTAAT |  |  |
| SS1G_03482F | ATATCAACGCCATCCAAT | Detection of the expression level of SS1G_03482 | 95 |
| SS1G_03482R | TCTATCAGCACGAATGTC |  |  |
| SS1G_12567F | ATCATCCAATACACCAGTC | Detection of the expression level of SS1G_12567 | 116 |
| SS1G_12567R | ATCACCAAACAACATCCA |  |  |
| SS1G_00900F | ACTTGAACGAGTCTAAGC | Detection of the expression level of SS1G_00900 | 143 |
| SS1G_00900R | AACCTTGCTCTTCTTCTC |  |  |
| SS1G_07597F | GGATTCAGCACCATACTT | Detection of the expression level of SS1G_07597 | 112 |
| SS1G_07597R | CTCGTAGATACCAGTTGTC |  |  |
| SS1G_10286F | GGCAAGTCAAGTTGTTAC | Detection of the expression level of SS1G_10286 | 168 |
| SS1G_10286R | CCTCTACTGTCATTCTCAAT |  |  |
| RACE3RT | CGATCGATCATGATGCAATGCNNNNNN | To obtain the viral cDNA | - |
| RACE-OLIGO: | GCATTGCATCATGATCGATCGAATTCTTTAGT GAGGGTTAATTGCC-(NH2) | Termini determination of genome | - |
| O5RACE -1 | GGCAATTAACCCTCACTAAAG |  |  |
| O5RACE -2 | TCACTAAAGAATTCGATCGATC |  |  |
| O5RACE -3 | CGATCGATCATGATGCAATGC |  |  |
